# Antithrombin-binding heparan sulfate is ubiquitously expressed in epithelial cells and suppresses pancreatic tumorigenesis

**DOI:** 10.1172/JCI184172

**Published:** 2025-09-16

**Authors:** Thomas Mandel Clausen, Ryan J. Weiss, Jacob R. Tremblay, Benjamin P. Kellman, Joanna Coker, Leo A. Dworkin, Jessica P. Rodriguez, Ivy M. Chang, Timothy Chen, Vikram Padala, Richard Karlsson, Hyemin Song, Kristina L. Peck, Satoshi Ogawa, Daniel R. Sandoval, Hiren J. Joshi, Gaowei Wang, L. Paige Ferguson, Nikita Bhalerao, Allison Moores, Tannishtha Reya, Maike Sander, Thomas C. Caffrey, Jean L. Grem, Alexandra Aicher, Christopher Heeschen, Dzung Le, Nathan E. Lewis, Michael A. Hollingsworth, Paul M. Grandgenett, Susan L. Bellis, Rebecca L. Miller, Mark M. Fuster, David W. Dawson, Dannielle D. Engle, Jeffrey D. Esko

**Affiliations:** 1 Department of Cellular and Molecular Medicine, UCSD, La Jolla, California, USA.; 2 John A. Burns School of Medicine, University of Hawaii, Honolulu, Hawaii, USA.; 3 Copenhagen Center for Glycomics, Department of Cellular and Molecular Medicine, Faculty of Health Sciences, University of Copenhagen, Copenhagen, Denmark.; 4 Department of Biochemistry and Molecular Biology and; 5 Complex Carbohydrate Research Center, University of Georgia, Athens, Georgia, USA.; 6 Salk Institute for Biological Studies, La Jolla, California, USA.; 7 Ragon Institute of Mass General Brigham, Massachusetts Institute of Technology, and Harvard, Cambridge, Massachusetts, USA.; 8 Biomedical Sciences Graduate Program and; 9 Medical Scientist Training Program, UCSD, La Jolla, California, USA.; 10 Pathology Residency Program, Warren Alpert Medical School of Brown University, Providence, Rhode Island, USA.; 11 Departments of Pediatrics and Bioengineering, UCSD, La Jolla, California, USA.; 12 Department of Pediatrics, Pediatric Diabetes Research Center, Sanford Consortium for Regenerative Medicine, UCSD, La Jolla, California, USA.; 13 Department of Pharmacology and Medicine, UCSD School of Medicine, La Jolla, California, USA.; 14 Cold Spring Harbor Laboratory, Cold Spring Harbor, New York, New York, USA.; 15 Division of Hematology-Oncology, University of Massachusetts Medical School, Worcester, Massachusetts, USA.; 16 The Bishop’s School, La Jolla, California, USA.; 17 Herbert Irving Comprehensive Cancer Center and Department of Physiology and Cellular Biophysics, Columbia University Medical Center, New York, New York, USA.; 18 Eppley Institute for Research in Cancer and Allied Diseases and; 19 Department of Internal Medicine, University of Nebraska Medical Center, Omaha, Nebraska, USA.; 20 Precision Immunotherapy, Graduate Institute of Biomedical Sciences, China Medical University, Taichung, Taiwan.; 21 Immunology Research and Development Center, China Medical University, Taiwan.; 22 Pancreatic Cancer Heterogeneity, Candiolo Cancer Institute – FPO – IRCCS, Candiolo, Turin, Italy.; 23 Department of Pathology, UCSD School of Medicine, La Jolla, California, USA.; 24 Department of Cell, Developmental and Integrative Biology, University of Alabama, Birmingham, Birmingham, Alabama, USA.; 25 Veterans Affairs San Diego Healthcare system, La Jolla, California, USA.; 26 UCSD division of Pulmonary, Critical Care, and Sleep Medicine, La Jolla, California, USA.; 27 Glycobiology Research and Training Center, UCSD, La Jolla, California, USA.; 28 Department of Pathology and Laboratory Medicine and Jonsson Comprehensive Cancer Center, David Geffen School of Medicine, UCLA, Los Angeles, California, USA.

**Keywords:** Cell biology, Oncology, Cancer, Coagulation, Glycobiology

## Abstract

3-*O*-sulfation of heparan sulfate (HS) is the key determinant for binding and activation of antithrombin III (AT). This interaction is the basis of heparin treatment to prevent thrombotic events and excess coagulation. Antithrombin-binding HS (HS^AT^) is expressed in human tissues but is thought to be expressed in the subendothelial space, mast cells, and follicular fluid. Here, we show that HS^AT^ is ubiquitously expressed in the basement membranes of epithelial cells in multiple tissues. In the pancreas, HS^AT^ is expressed by healthy ductal cells, and its expression is increased in premalignant pancreatic intraepithelial neoplasia lesions but not in pancreatic ductal adenocarcinoma (PDAC). Inactivation of *HS3ST1*, a key enzyme in HS^AT^ synthesis, in PDAC cells eliminated HS^AT^ expression, induced an inflammatory phenotype, suppressed markers of apoptosis, and increased metastasis in an experimental mouse PDAC model. HS^AT^-positive PDAC cells bind AT, which inhibits the generation of active thrombin by tissue factor and factor VIIa. Furthermore, plasma from patients with PDAC showed accumulation of HS^AT^, suggesting its potential as a marker of tumor formation. These findings suggest that HS^AT^ exerts a tumor-suppressing function through recruitment of AT and that the decrease in HS^AT^ during progression of pancreatic tumorigenesis increases inflammation and metastatic potential.

## Abstract 2

Antithrombin III binding heparan sulfate is expressed in epithelial cells and has a tumor suppressing function in pancreatic cancer precursor lesions.

## Graphical abstract

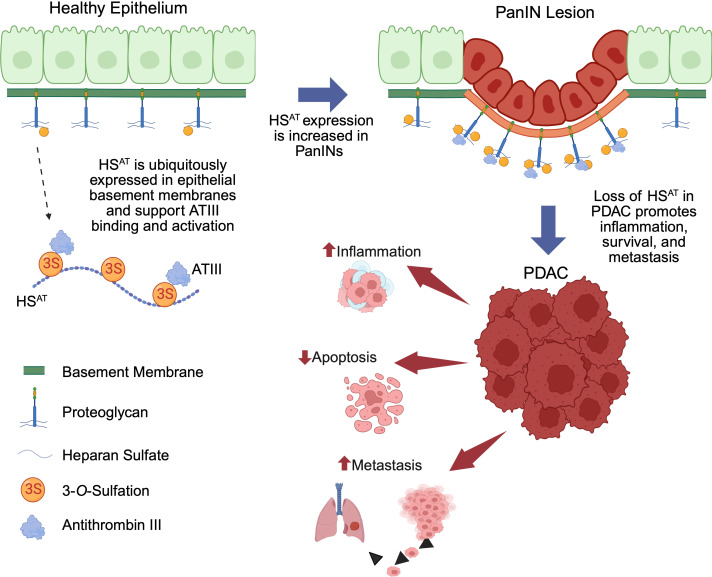

## Introduction

**Figure 1:**
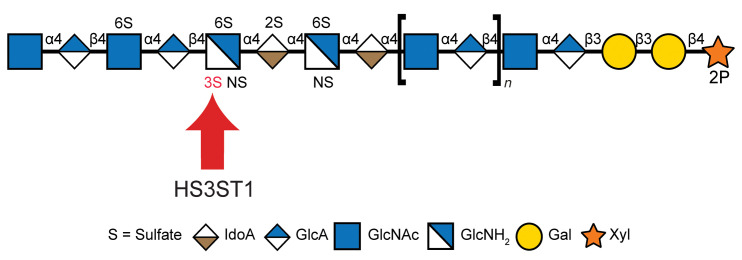
Representation of heparan sulfate structure. Illustration uses the symbol nomenclature for glycans (71) and shows where the 3-*O*-sulfate group is added by HS3ST1.

Glycosaminoglycans are linear polysaccharides consisting of alternating amino sugars and uronic acid residues, covalently attached to the core proteins of proteoglycans in the extracellular matrix (ECM) and plasma membrane (1). The human genome encodes 17 primary heparan sulfate (HS) proteoglycans and several less-abundant “part-time” proteoglycans. HS polysaccharides from different tissues have different compositions of disaccharide subunits, operationally generated by cleavage of the chains with bacterial heparin lyases or nitrous acid (2). The basic disaccharide consists of β1-4 linked d-glucuronic acid and α1-4 linked *N*-acetyl-d-glucosamine residues (Figure 1). During the assembly process, clusters of these disaccharides undergo processing reactions in which some *N*-acetyl-d-glucosamine residues become *N*-deacetylated and *N*-sulfated; adjacent glucuronic acid units undergo epimerization at carbon-5 to become l-iduronic acid; and ester-linked sulfate groups are installed at the 2-hydroxyl group of the uronic acids and the 6-hydroxyl group and, more rarely, at the 3-hydroxyl group of the glucosamine residues. In general, these modification reactions do not go to completion, giving rise to domains of the chains with variably sulfated sugars interspersed by domains lacking all or most of these modifications. The variable length of the sulfated and nonsulfated domains and their pattern of *N-* and *O-*sulfation create unique motifs that make up binding sites for HS-binding proteins. Binding can lead to altered growth factor signaling, endocytosis, lipoprotein uptake, and other essential cell biological and physiological responses (3).

Heparin is one of the best studied forms of HS. Heparin and its low molecular weight derivatives are potent anticoagulants effective in the prevention and treatment of thrombotic events, such as deep vein thromboses and pulmonary emboli, and excess coagulation during surgical procedures (4). The primary mechanism of action depends on heparin’s interaction with antithrombin III (AT, SERPINC1), which enhances the ability of AT to inhibit primarily factor II (thrombin) and factor Xa in the coagulation cascade. AT binding to heparin induces a conformational change in AT and extension of the reactive loop, which can then covalently inactivate serine proteases involved in the coagulation cascade (5, 6). Heparin is a highly sulfated form of HS, and it is generated by fractionation of glycosaminoglycans derived from pig entrails or bovine lung. Compared with HS, heparin is highly enriched in sulfate groups, iduronic acid, and the rare pentasaccharide sequence that binds to and activates AT (6–8). The active pentasaccharide carries sulfate groups at several locations, but sulfation at the 3-hydroxyl group of glucosamine residues is a key determinant of activity (7–9) (Figure 1, marked in red). The primary enzyme responsible for the addition of this 3-*O*-sulfate group is called HS3ST1 (10), and the HS product of HS3ST1 binds to AT and is termed HS^AT^. Although HS is ubiquitously produced by all vertebrate and invertebrate cells, HS^AT^ in healthy tissue is much less prevalent (11–17). Heparin is thought to be exclusively expressed in granulated connective tissue type mast cells through unclear regulatory mechanisms (18, 19).

The expression of HS proteoglycans and the composition of their attached HS chains is abnormal in many types of tumors (20). In general, one of the most frequent changes in cancer cells is the composition of cell surface glycans, which modulate tumor cell interactions with the surrounding environment (21, 22). Glycosaminoglycans regulate key processes in cancer, such as tumor cell proliferation, adhesion, migration, invasion, metastasis, and angiogenesis (23, 24). Thus, insights into specific alterations in the cancer-specific composition of glycosaminoglycans and how such alterations mediate cancer progression could lead to the discovery of biomarkers and targets for anticancer therapy.

Pancreatic ductal adenocarcinoma (PDAC) is the most common form of pancreatic cancer and one of the most fatal malignancies, with a 5-year survival rate of approximately 13% (25). A distinguishing characteristic that contributes substantially to the poor outcome of PDAC is a dense, fibrotic ECM with a highly elevated content of glycosaminoglycans, specifically hyaluronic acid, chondroitin sulfate, and HS (26). The high glycosaminoglycan content has been linked to a general noninflammatory tumor phenotype and limits the accessibility of chemo- and immunotherapeutic drugs (26–30). PDAC cells specifically overexpress multiple HS proteoglycans, including glypican-1 (31) and glypican-3 (32). A recent study found that 3 HS proteoglycans were among the most highly overexpressed cell surface proteins modulated by *KRAS-*activating mutations in PDAC compared with normal pancreatic tissue (33). Moreover, glypican-1–positive exosomes in serum can distinguish patients with different stages of pancreatic cancer and benign pancreatic disease with high specificity and sensitivity (34). Unfortunately, glypican-1 and other HS proteoglycans are expressed in normal tissues as well and can be shed into the circulation as a result of inflammation or infection (35–37), making it difficult to use these HS proteoglycans for early detection.

Here, we show that HS^AT^ is expressed in epithelial layers across multiple organs. *HS3ST1* gene expression is elevated in PDAC, and the resulting HS^AT^ supports binding and activation of AT. In accordance with this finding, we show that HS^AT^ can be detected in premalignant lesions and PDAC, and in the plasma from patients with pancreatic cancer. HS^AT^-positive tumors bind exogenously added and endogenously produced AT. Bound AT can regulate tumorigenic signaling and inflammation, in part by inhibiting the activity of tissue factor–initiated (TF-initiated) generation of factor Xa and thrombin. Murine PDAC cells deficient in HS^AT^ showed increased metastasis, an inflammatory phenotype, and reduced markers of apoptosis in vivo. We propose a model in which expression of HS^AT^ has a tumor-suppressing function through recruitment of AT. Fully understanding the abundance and function of HS^AT^ and its bound AT could potentially pave the way for new intervention strategies in PDAC.

## Results

### HS^AT^ is ubiquitously expressed by epithelial cells across many organs.

**Figure 2:**
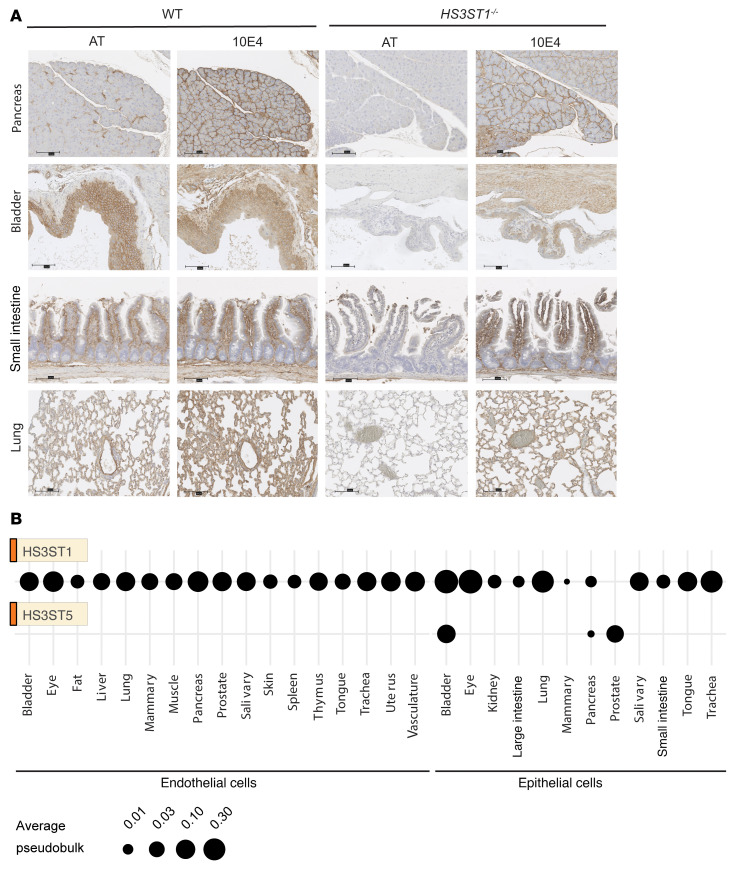
HS^AT^ is expressed by epithelial cells across different organs. (**A**) AT staining of HS^AT^ in FFPE tissue sections from various organs isolated from WT and *HS3ST1*^–/–^ mice. mAB 10E4 was used to stain for general HS. HS^AT^ staining occurred on epithelial cells across all tested organs and was dependent on expression of *HS3ST1*. Effect of HSase treatment is shown in Supplemental Figure 1. Scale bar: 100 μm. (**B**) Analysis of *HS3ST1* and *HS3ST5* mRNAs in multiple human organs (data obtained from the Tabula Sapiens; ref. 39). *HS3ST1* expression occurred across endothelial and in epithelial cells, with the exception of the prostate. *HS3ST5* expression was more restricted.

HS^AT^ expression in human tissues has previously been thought to be somewhat rare (11–17). To further map HS^AT^ expression across organs, we developed a staining protocol based on binding of exogenous human AT to tissue sections. Surprisingly, staining for HS^AT^ was observed in the epithelial compartment in tissues across multiple mouse organs, including the pancreas, bladder, small intestine, and lung (Figure 2A). Systemic genetic inactivation of *HS3ST1* in the mouse (38) abrogated AT binding, illustrating the specificity of AT staining for 3-*O*-sulfated HS generated by the HS3ST1 isozyme. Staining with mAb 10E4, which binds to generic HS, showed general HS expression throughout the tissues and as expected was insensitive to inactivation of *HS3ST1*. Similar results were obtained across other tissues (Supplemental Figure 1A; supplemental material available online with this article; https://doi.org/10.1172/JCI184172DS1). Interestingly, inactivation of *HS3ST1* only partially abrogated AT binding in the stomach, with residual AT staining present in the luminal gastric mucosa (Supplemental Figure 1A). Although HS3ST1 may be the main enzyme for HS^AT^ expression in stomach tissue, another isoenzyme capable of forming the AT pentasaccharide (e.g., HS3ST5) may be expressed in the luminal gastric mucosa. Treatment of the tissue sections with heparin lyase (HSase), which digests HS, blocked binding of both AT and mAb 10E4, further supporting the specificity of the staining methods (Supplemental Figure 1B).

The discovery of widespread expression of HS^AT^ in epithelial cells was surprising because low expression of *HS3ST1* has been reported across tissues (17). To further investigate this observation, we analyzed expression patterns of *HS3ST1* mRNA in epithelial cells in various organs using the Tabula Sapiens resource (39). Except for the prostate gland, widespread expression of *HS3ST1* was noted, in accordance with the HS^AT^ staining (Figure 2B). *HS3ST5* mRNA, another HS3ST isozyme capable of producing HS^AT^ (40), was highly expressed in prostate and bladder epithelial cells and to a lesser extent in pancreatic epithelial cells. Endothelial cells from all the organs expressed moderate levels of *HS3ST1*, consistent with the known expression of HS^AT^ (17). These findings challenge the generally accepted notion that HS^AT^ is an endothelial cell–specific marker and demonstrate widespread expression by epithelial cells (17).

### HS^AT^ is specifically expressed by ductal cells in the healthy pancreas.

**Figure 3:**
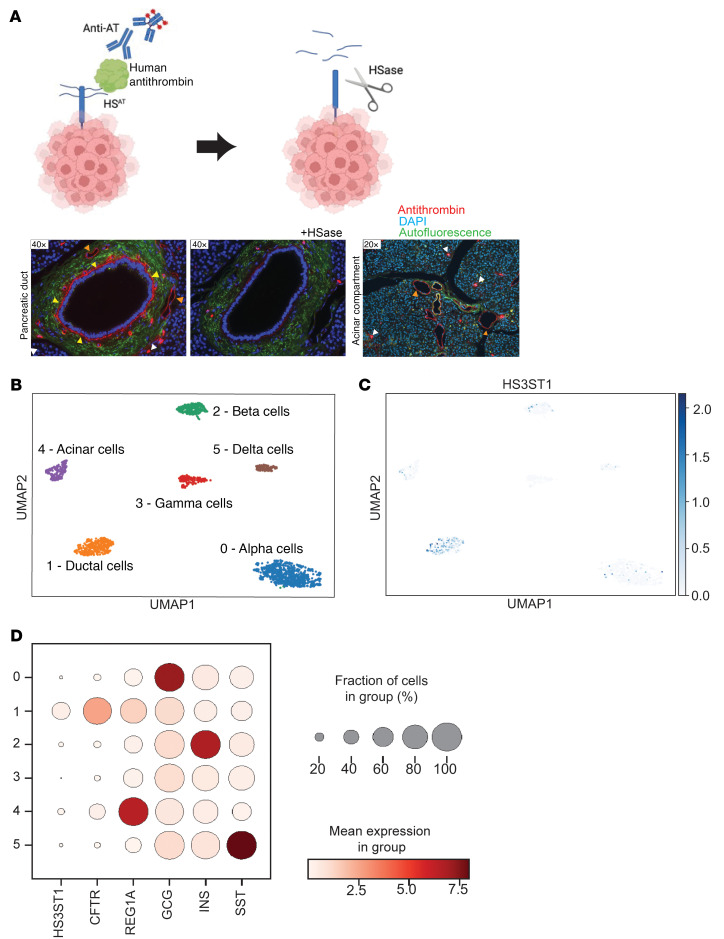
HS^AT^ is expressed by ductal cells in the healthy pancreas. (**A**) The graphic illustrates the staining procedure and the effect of HSase treatment. AT staining of FFPE section of human pancreatic tissue, with and without heparin lyase (HSase) treatment. AT staining was intense along the basolateral side of the normal ductal cells (yellow arrowheads) and blood vessels (orange arrowheads). The image on the right displays the acinar compartment. AT staining is seen in the blood vessels as expected (orange arrowheads) and occasional cells in the stroma (white arrowheads). Bound AT, red; DAPI-stained nuclei blue; autofluorescence, green. (**B**–**D**) Analysis of *HS3ST1* mRNA expression in a single-cell sequence data set from healthy pancreas. (**B** and **C**) Uniform Manifold Approximation and Projection (UMAP) visualization of single-cell RNA-Seq clusters based on markers identifying acinar, beta, alpha, delta, and ductal cell compartments. Each point represents a single cell, and cells are positioned based on similarity in gene expression. (**D**) *HS3ST1* mRNA expression in the identified clusters. The level of expression of *HS3ST1* and known cell markers (CFTR [ductal], REG1A [acinar], GCG [alpha cells], INS [beta cells], and SST [delta cells]) are shown. *HS3ST1* expression is highest in the ductal cell fraction.

To further investigate HS^AT^ expression in the pancreas, we obtained tissue samples from healthy human pancreas and stained them for HS^AT^ by immunofluorescence using human AT and a mAb to AT (Figure 3A). Very specific staining on the basolateral side and basement membrane of the epithelial ductal cells lining the pancreatic ducts was observed (yellow arrowheads, Figure 3A). In contrast, the exocrine acinar compartment did not stain (Figure 3A). The endothelial lining of the pancreatic vasculature (orange arrowheads, Figure 3A) stained as well, consistent with previously published reports of HS^AT^ expression in endothelial cells (17) (Figure 3A). Interestingly, we also found individual cells of high expression throughout the pancreatic parenchyma (white arrowheads, Figure 3A). Mast cells have previously been described to produce HS^AT^, but whether the occasional cells that stained strongly with AT are mast cells will require further investigation (41).

To corroborate these findings and to further investigate the origin of the HS^AT^ expression in the pancreas, we examined *HS3ST1* expression in a single-cell sequencing data set from healthy human pancreas (42). Specific cell clusters were identified by expression of cell-specific markers, such as cystic fibrosis transmembrane conductance regulator (*CFTR*), which regulates secretion of digestive enzymes in ductal cells; regenerating islet-derived protein 1 alpha (*REG1A*), which is expressed in acinar cells and is involved in pancreatic regeneration; glucagon (*GCG*), which is produced by alpha cells in the pancreas and regulates blood sugar levels; insulin (*INS*), which is produced by beta cells in the pancreas and is critical for regulating glucose metabolism; and somatostatin (*SST*), which is expressed by pancreatic delta cells. *HS3ST1* expression correlated with markers of ductal cells (Figure 3, B–D) and had limited expression in the other pancreatic cell types. These findings show that HS^AT^ is expressed by pancreatic ductal cells and that it accumulates in the basement membrane on the basolateral side of the pancreatic ducts.

### HS3ST1 is upregulated in pancreatic cancer and is specifically expressed by malignant ductal-like cells.

**Figure 4:**
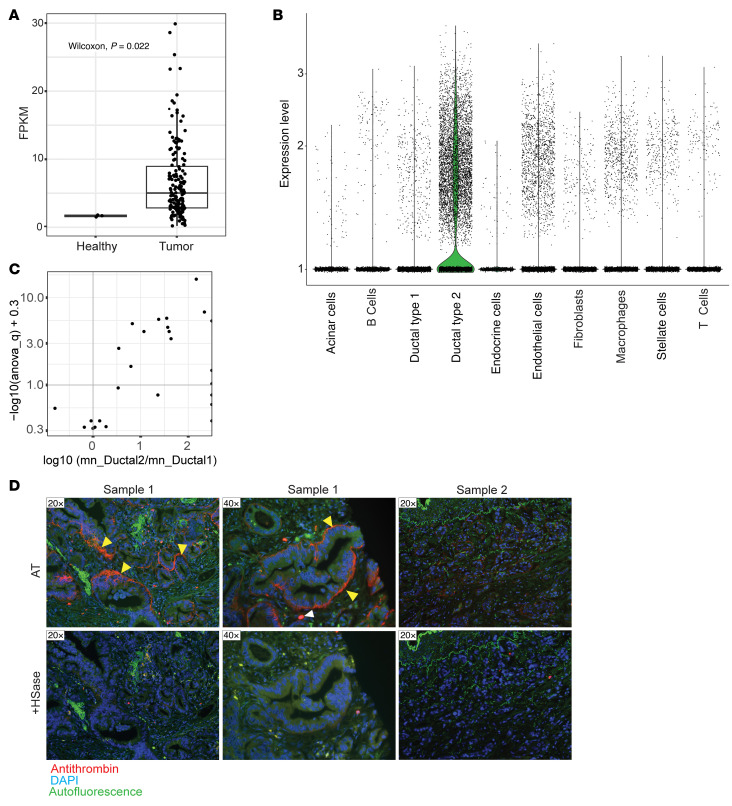
*HS3ST1* mRNA expression is upregulated in PDAC. (**A**) *HS3ST1* mRNA expression across tumor stages in PDAC in the TCGA-PAAD data set. Statistical analysis by Wilcoxon rank-sum test. (**B**) *HS3ST1* mRNA expression in single-cell RNA-Seq data set from Peng et al. (43). HS3ST1 transcripts are primarily expressed in the ductal cell type 2 cluster, associated with malignant ductal-like cells. (**C**) Single-cell *HS3ST1* mRNA expression across human PDAC samples integrated across multiple cohorts (44). Scatter plot showing log-ratio of median expression by subject in type-2 and type-1 ductal cells stratified by inverse-log FDR of 1-way ANOVA-computed *P* values comparing expression in type-2 and type-1 ductal cells. (**D**) Sample 1: AT staining of FFPE sections of human PDAC tissue, with and without HSase treatment. AT stained the basolateral side of the polarized PDAC cells (yellow arrowheads) and was HS dependent. Some cells in the stroma, most likely mast cells, stained strongly due to the presence of heparin-like material (white arrowhead). Sample 2: AT staining of FFPE sections from healthy human pancreas. Bound AT, red; DAPI-stained nuclei blue; autofluorescence, green.

A survey of *HS3ST1* mRNA expression in the Human Protein Atlas Database (https://www.proteinatlas.org/) showed that pancreatic ductal adenocarcinoma (PDAC) cancer cell lines express *HS3ST1* transcripts. To investigate the clinical significance of this observation, we examined *HS3ST1* expression across The Cancer Genome Atlas (TCGA) Pancreatic Adenocarcinoma Database (PAAD) data set, which is publicly available (https://portal.gdc.cancer.gov/projects/TCGA-PAAD). A significant increase in *HS3ST1* mRNA expression was observed between healthy bulk pancreatic tissue and tumors in this data set (Figure 4A). The low expression in healthy pancreas in this data set could be due to the low number of ductal cells in bulk healthy tissue, compared with the number of ductal-like cells found in tumor tissue. To investigate the source of HS^AT^ expression in PDAC, we analyzed the cell-specific expression of *HS3ST1* in the single-cell sequencing data set reported by Peng et al. (43). *HS3ST1* was strongly expressed in the ductal type 2 cell cluster, which was reported to represent the ductal-like malignant cells within the data set (Figure 4B) (43). Interestingly, this data set did not show much *HS3ST1* expression in the ductal type 1 cluster, which was reported to represent healthy ductal cells. A direct comparison of *HS3ST1* expression in healthy versus malignant ductal-like cells in individual patients, reported in the PDAC reference single-cell RNA-Seq data set by Chijimatsu et al., showed a significantly higher *HS3ST1* expression in malignancy in most samples (Figure 4C) (44).

Staining of primary PDAC patient tumor biopsies revealed that HS^AT^, like healthy pancreatic ducts, was concentrated on the basolateral side and basement membrane of the polarized tumor cells within ductal-like tumor lesions (yellow arrowheads, Figure 4D). The staining was specific for HS^AT^ given that treatment with HSase completely abrogated AT binding. Areas of tumor not associated with a ductal-like phenotype showed little to no staining (Sample 2, Figure 4D), suggesting that *HS3ST1* expression correlates with the ductal cell phenotype. The discovery of HS^AT^ in pancreatic cancer is interesting because acinar cells that do not express *HS3ST1* have been suggested to be one possible source of malignant ductal-like cells, although the exact cell of origin is still debated (45). Taken together, these data show that *HS3ST1* and HS^AT^ are expressed in PDAC.

### HS^AT^ is expressed in early pancreatic intraepithelial neoplasia lesions of PDAC development and correlates with favorable outcome.

**Figure 5:**
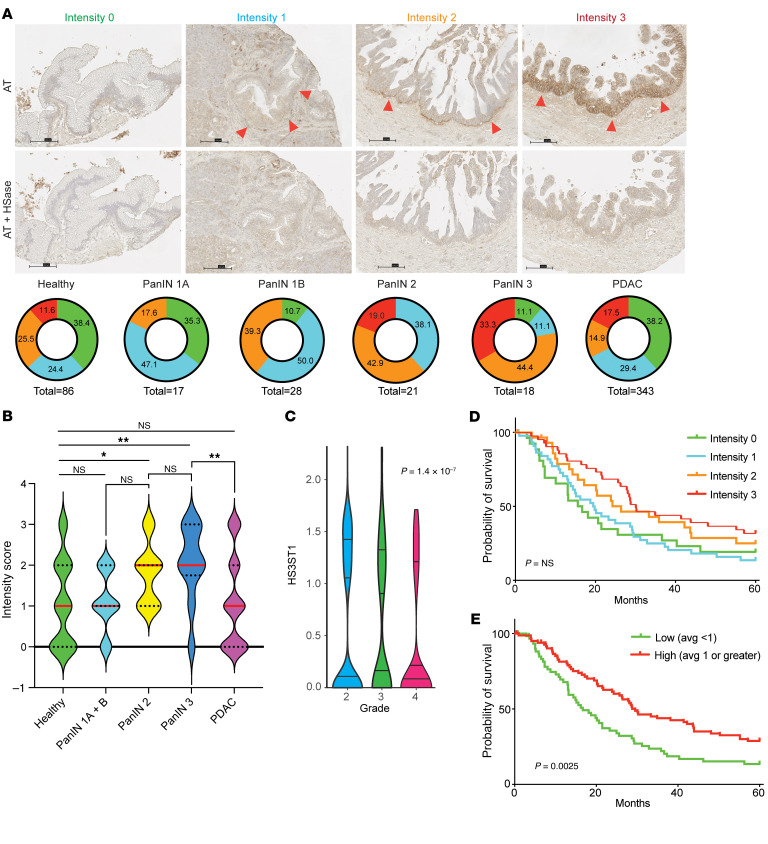
HS^AT^ is expressed in early pancreatic intraepithelial neoplasia lesions leading to PDAC and correlates with favorable outcome. (**A**) Representative cores from a TMA containing FFPE triple cores from 150 patients with PDAC, including dissected pancreatic intraepithelial neoplasia (PanIN) lesions and healthy adjacent pancreatic tissue, stained for HSAT. AT staining was intense along basolateral side of tumor lesions (red arrowheads). Tissue was scored based on staining intensity: 0, less than 10% membrane staining; 1, faint/barely perceptible basolateral or complete membrane staining in 10% or more of tumor core; 2, weak basolateral or complete membrane staining in 10% or more of tumor core; 3, moderate/strong basolateral or complete membrane staining in 10% or more of tumor core. In representative images, intensities 0 and 1 are low-grade PanIN lesions; 2 and 3 are high-grade PanIN lesions. Pie charts show cumulative grading intensity 0 (green), 1 (blue), 2 (orange), or 3 (red) of the entire TMA (individual cores included separately). Removal of HS with heparin lyase diminished staining. . Numbers represent percentage of cores with a given intensity stain. Scale bar: 100 μm. (**B**) Quantification of data in **A**. An increase in HS^AT^ seen during PanIN progression compared with healthy ducts. No significant increase seen in PDAC versus healthy pancreas. Median (red line) and quartiles (dotted lines) are illustrated; 1-way ANOVA with Tukey’s multiple-comparison correction. *P ≤ 0.05; **P ≤ 0.01; NS, not significant. (**C**) Correlation between HS3ST1 expression and tumor grade in PDAC reference single-cell RNA-Seq data set (44). (**D**) Association between HS^AT^ expression in PDAC samples and patient survival (PanIN lesions excluded and intensity of HS^AT^ staining included as average of 3 patient-specific tissue cores). A stepwise improvement in survival with increased HS^AT^ was evident but did not achieve significance. (**E**) Association between HS^AT^ expression and patient survival for high versus low HS^AT^ stain. Presence of HS^AT^ was significantly associated with better outcome; log-rank (Mantel-Cox) test.

To elucidate whether the increased expression of *HS3ST1* results in elevated expression of HS^AT^ in pancreatic cancer, we stained a 140-patient tissue microarray (TMA) of treatment-naive, early-stage PDAC — stages I and II as defined by the American Joint Committee on Cancer (AJCC) 7th edition. The TMA included 3 separate cores for each patient tumor, as well as patient-matched, premalignant, pancreatic intraepithelial neoplasia lesions (PanIN) (46). AT staining was scored by one of the authors, a practicing subspecialty gastrointestinal pathologist. A significant increase in the intensity of staining was noted in high-grade PanIN compared with healthy pancreas (Figure 5, A and B). Interestingly, a reduction in AT staining was seen between PanIN3 and PDAC. No significant difference was seen between HS^AT^ staining in healthy pancreas and PDAC. This result deviates from the *HS3ST1* mRNA expression shown in Figure 4A and likely reflects the difference in analysis of bulk tumor containing a mix of precursor and PDAC cells (Figure 4A) versus grade-specific expression at the cellular level (Figure 5, A and B) and differences in RNA expression versus HS3ST1 expression. Treatment of serial sections with HSase confirmed that the staining was dependent on HS (Figure 5A). A similar drop was seen in *HS3ST1* mRNA expression in high-grade tumors in the PDAC reference single-cell RNA-Seq data set (Figure 5C) (44). Analysis of multivariate proportional hazards of the PDAC cohort revealed a significant negative correlation between high AT (average score ≥1) and risk of death in the patient group (HR 0.67; 95% CI, 0.45–0.99) (*P* < 0.048) (Table 1). Univariate Cox analysis of overall survival showed that high AT staining (average score ≥1) had an adjusted HR of 0.56 (95% CI, 0.38–0.82) (*P* = 0.003) (Table 2). AT staining was significantly associated with low histological grade (*P* < 0.001, χ^2^ test) and lower AJCC stage (*P* = 0.009, χ^2^ test) but was not correlated with a variety of other clinicopathological variables (Table 3). Kaplan-Meier analysis also showed a trend between increased survival and higher HS^AT^ staining, with a significant difference between patients exhibiting high staining or low staining (median survival 23.5 months [95% CI, 18.8–28.2] for low AT staining [average score <1] and 34.3 months [29.8–38.8] for high AT staining [average score ≥1]; log-rank test *P* = 0.002) (Figure 5, D and E). Further analysis revealed a strong correlation between HS^AT^ staining and low histological grade as well as with stage I versus II (Table 3).

### Murine PDAC cells express HS^AT^ dependent on HS3ST1.

**Figure 6:**
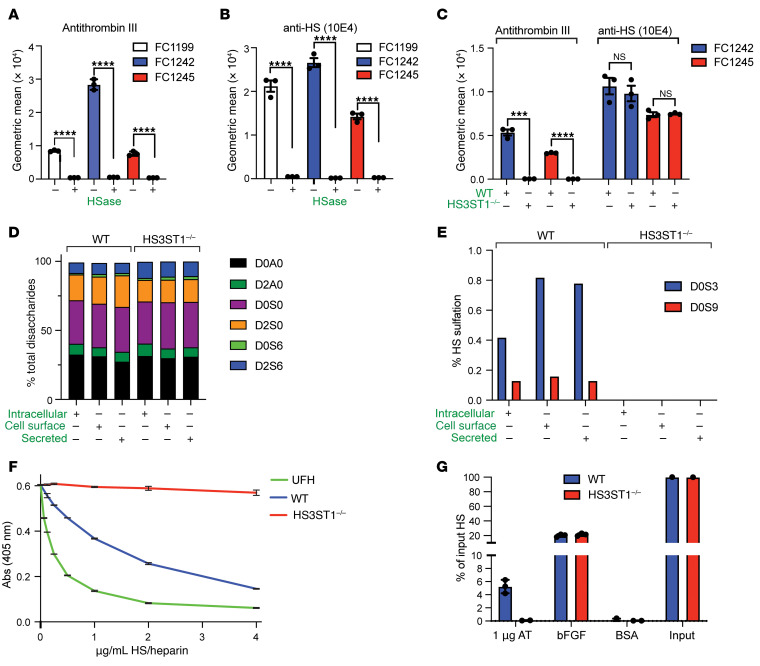
Murine PDAC cells derived from the *Kras*^LSL.G12D/+^ *p53*^R172H/+^ *Pdx*^Cre^ (KPC) mouse express HS^AT^. (**A**) AT bound to FC1199, FC1242, and FC1245 as measured by flow cytometry. Treatment with heparin lyase (HSase) abrogated AT binding. Graph shows mean ± SEM. (**B**) mAB 10E4 (anti-HS) binding to FC1199, FC1242, and FC1245 by flow cytometry. HSase treatment diminished mAb binding. Graph shows mean ± SEM. (**C**) Genetic inactivation of *HS3ST1* in FC1242 and FC1245 abolished AT binding but had no effect on binding of mAb 10E4. Graph shows mean ± SEM. (**D**) Disaccharide analysis of purified HS from WT FC1242 and *HS3ST1*^–/–^ cells. (**E**) 3-*O*-sulfated disaccharides (D0S3 and D0S9) were present in FC1242 cells. No 3-*O*-sulfation was detected in *HS3ST1*^–/–^ cells. (**F**) Anti-FXa assay was used to determine the presence of HS^AT^ in purified HS from WT and *HS3ST1*^–/–^ FC1242 cells compared with unfractionated heparin (UFH). (**G**) Binding of [^35^S]HS to AT and FGF-2 as measured by filtration across nitrocellulose. Approximately 5% of input [^35^S]HS from WT FC1242 HS cells bound AT, whereas [^35^S]HS from *HS3ST1*^–/–^ cells did not. BSA served as a negative control. Statistical analysis by 2-tailed *t* test in **A**–**C**. ***P ≤ 0.001; ****P ≤ 0.0001; NS, not significant.

To investigate the role of HS^AT^ expression in PDAC in a model organism, we used 3 murine tumor cell lines (FC1199, FC1242, and FC1245; ref. 47) derived from the *Kras*^LSL.G12D/+^ *p53*^R172H/+^ *Pdx*^Cre^ (KPC) mouse model of pancreatic cancer (48). All 3 lines stained positive for HS^AT^ (Figure 6A) and mAb 10E4 (Figure 6B) in flow cytometry, and treatment of the cells with HSase completely abolished binding. Cell staining showed a uniform granular membrane staining pattern (Supplemental Figure 2A), suggesting that the basolateral HS^AT^ staining seen in tissue (Figure 4D) is a product of cellular polarization as occurs in complex tissues that is not manifested in monolayer cultures. Inactivation of *HS3ST1* using CRISPR/Cas9 (Supplemental Figure 2B) eliminated AT binding without affecting overall HS expression, as assessed by staining with mAb 10E4 (Figure 6C).

Analysis of cell surface (trypsin-releasable), secreted (in conditioned serum-free cell media), and intracellular (cell pellet) HS showed no major differences between WT cells and *HS3ST1*^–/–^ in disaccharide composition (Figure 6D and Supplemental Figure 3) and little variation in the proportion in different fractions (Supplemental Figure 3). The amount of 3-*O*-sulfated disaccharides released by exhaustive digestion with HSase was generally low but could be detected using a recently described HPLC-based method (49). WT cells mainly produced D0S3 (∆^4,5^UA-GlcNS3S) and D0S9 (∆^4,5^UA-GlcNS3S6S) (Figure 6E and Supplemental Figure 3). These 3-*O*-sulfated disaccharides were completely absent in the *HS3ST1*^–/–^ lines, indicating that the HS3ST1 isozyme was responsible for their generation in these cells.

In blood, binding of anticoagulant heparin to AT greatly accelerates the inhibition of serine proteases in the coagulation cascade, primarily factor Xa and factor II (thrombin). To test whether PDAC-derived HS could activate AT, purified HS from the cells was added to the standard anti-FXa assay that is used clinically to monitor plasma heparin in patients (50, 51). Purified PDAC HS showed potent activity with an IC_50_ value of 1.9 μg/mL (95% CI, 1.7–2.1). This was approximately 8-fold higher than the value obtained for unfractionated heparin (IC_50_ 0.25 μg/mL; 95% CI, 0.23–0.28) (Figure 6F). AT activation was completely abolished by genetic inactivation of *HS3ST1*.

To estimate the preponderance of HS^AT^ chains in PDAC HS, cells were grown in media containing [^35^S]O_4_, which cells incorporate into HS. A mixture of [^35^S]HS and AT was then filtered rapidly through a nitrocellulose membrane (52). Free [^35^S]HS passes through the membrane, but [^35^S]HS bound to AT is retained, and the ratio indicates the proportion of HS chains that carry the HS^AT^ determinant. About 5% of the HS chains bound to AT by this assay, whereas [^35^S]HS derived from *HS3ST1*^–/–^ cells did not bind (Figure 6G). Approximately 20% of [^35^S]HS from WT and *HS3ST1*^–/–^ cells bound to FGF-2, which binds HS independently of HS3ST1-mediated 3-*O*-sulfation.

### HS^AT^ circulates in plasma from patients with pancreatic cancer and pancreatitis.

HS is expressed on cell surfaces and in the ECM and circulates in bodily fluids (53). Having shown that HS^AT^ from murine KPC cells is secreted into the cell culture medium (Figure 6, D and E) and that this HS^AT^ can be detected using the clinical anti-FXa assay (Figure 6F), we tested whether HS^AT^ could be measured in the plasma from patients with pancreatic cancer. HS was purified by anion exchange chromatography from flash frozen plasma from patients with PDAC, pancreatitis, and intraductal papillary mucinous neoplasms. The purified HS was then tested for the presence of HS^AT^ by measuring its ability to activate AT and cause inhibition of FXa (Supplemental Figure 4). Varying amounts of anti-Xa HS^AT^ activity were measured in all patients with pancreatic cancer but not in pooled plasma from healthy individuals. This activity was completely abrogated by pretreating the purified HS with HSase, supporting the specificity of the assay. HS^AT^ activity was also found in patients with pancreatitis, suggesting that release of HS^AT^ is not specific to cancer and could be released from healthy ducts due to other insults such as inflammation (Supplemental Figure 4).

### Loss of HS^AT^ increases tumor inflammation and metastasis.

**Figure 7:**
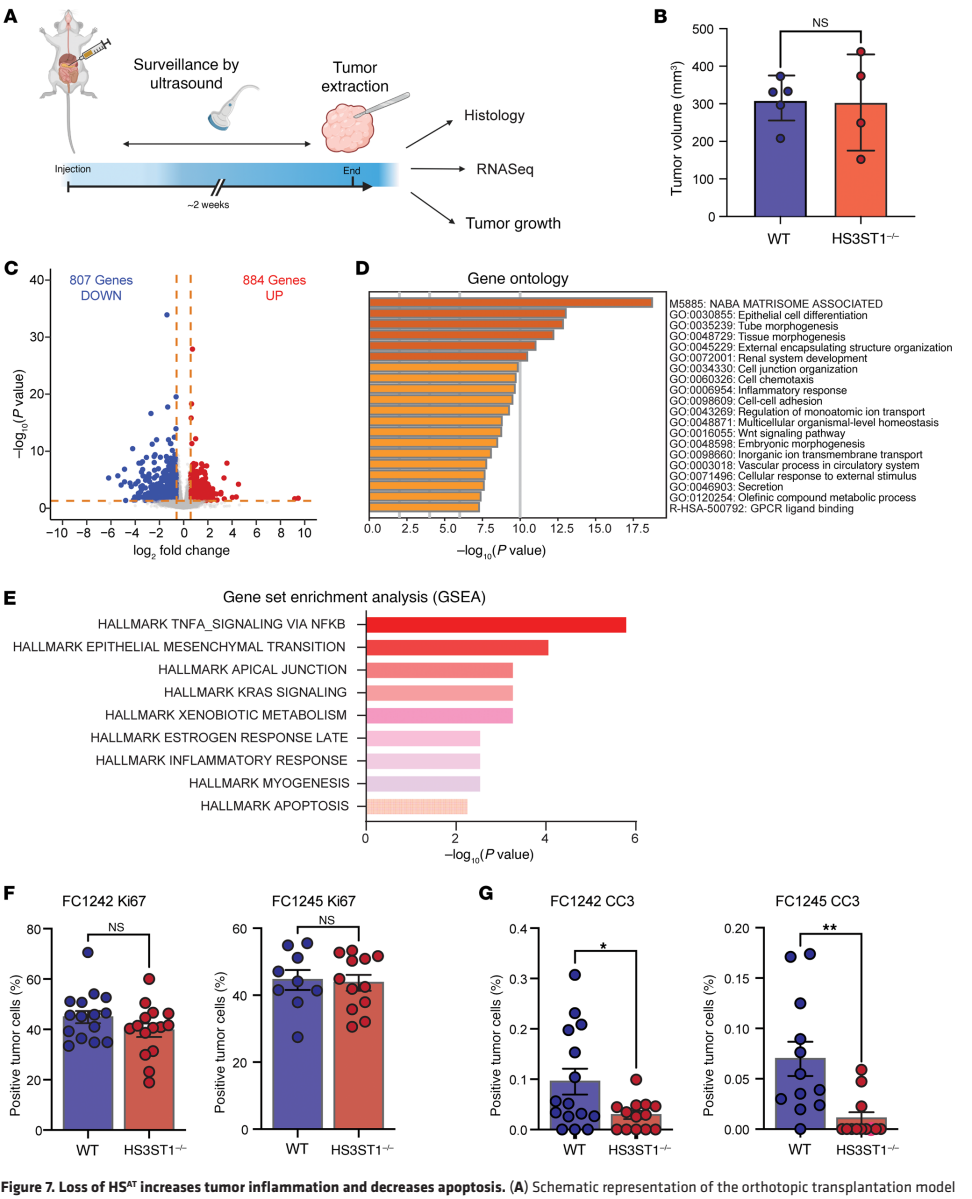
Loss of HS^AT^ increases tumor inflammation and decreases apoptosis. (**A**) Schematic representation of the orthotopic transplantation model performed with WT and *HS3ST1*^–/–^ F1242 and FC1245 lines. Graphic illustration created in BioRender (https://BioRender.com/x05fpme). (**B**) Tumor size at 2 weeks after implantation, measured by ultrasound. No significant difference in tumor size observed. (**C**) RNA was extracted from the tumors (*n* = 5 per group) and subjected to RNA-Seq analysis. Data represented as a volcano plot with cutoffs of adjusted *P* value < 0.05 and log_2_ fold change ≥ 0.58. (**D**) Gene ontology analysis was analyzed in the upregulated genes in *HS3ST1*^–/–^ line (log_2_ fold change > 0.58, adjusted *P* < 0.05) using Metascape (54). (**E**) Gene Set Enrichment Analysis was performed on the same data set, revealing enrichment for pathways involved in inflammation, EMT, and KRAS signaling. (**F** and **G**) Staining for Ki67 in FFPE tissue sections derived from the tumors (**F**) and for cleaved caspase-3 (CC3) (**G**). Graphs show the mean ± SD. Statistical analysis by 2-tailed *t* test in **B**, **F**, and **G**. *P ≤ 0.05; **P ≤ 0.01; NS, not significant.

To investigate the activity of HS^AT^ in tumorigenesis, murine PDAC cells were transplanted orthotopically into the pancreas of syngeneic mice (Figure 7A). Ultrasound imaging showed no significant difference in overall tumor size between WT and *HS3ST1*^–/–^ tumors 2 weeks after implantation (Figure 7B). Postmortem histology showed that the tumors derived from WT cells expressed HS^AT^, as expected, whereas tumors derived from *HS3ST1*^–/–^ cells did not (Supplemental Figure 5A). Interestingly, sections from *HS3ST1*^–/–^ tumors showed some focal HS^AT^ staining in the tumor microenvironment (Supplemental Figure 5A, red arrows). This staining could reflect binding of AT to low-affinity HS binding sites, selective expression of HS3ST5, or infiltration by HS^AT^-producing cells. RNA-Seq of WT and *HS3ST1*^–/–^ tumors derived from both FC1242 and FC1245 cells (*n* = 5 each group, 20 tumors total) revealed significant alterations in gene expression (Figure 7C). Gene ontology analysis of genes upregulated in *HS3ST1*^–/–^ cells using Metascape (54) revealed significant alterations in multiple pathways involved in matrisome formation, cellular epithelial cell differentiation, and tissue morphogenesis (Figure 7D). Gene set enrichment analysis (GSEA) of upregulated genes showed enrichment for pathways involved in inflammation, epithelial-mesenchymal transition (EMT), KRAS signaling, and apoptosis (Figure 7E). Staining for Vimentin, a marker of EMT, was indeed significantly increased in *HS3ST1*^–/–^ tumors (Supplemental Figure 5B), suggesting that HS^AT^ is related to EMT. AT binding to a murine cell line derived from a tumor that developed in mice with p53 deletion combined with a doxycycline-inducible mutant KRasG12D (iKRAS) (55) showed an increase in staining upon induction of KRAS expression with doxycycline (Supplemental Figure 5C), suggesting a link between KRAS status and HS^AT^ expression as previously described for several HS proteoglycans (33). Finally, no difference was observed in staining of the tumors for the proliferation marker Ki67 (Figure 7F), whereas a significant difference was noted in apoptotic activity, with *HS3ST1*^–/–^ tumors staining significantly lower for cleaved caspase-3 (Figure 7G). However, these differences did not manifest in a significant difference in tumor growth in the cohort.

**Figure 8:**
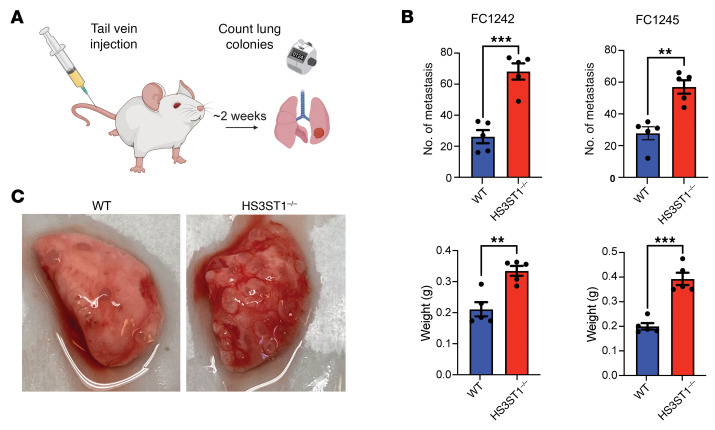
Loss of HS^AT^ increases tumor metastatic seeding capacity. (**A**) Schematic representation of the model for experimental metastasis after intravenous injection of WT and *HS3ST1*^–/–^ F1242 and FC1245 PDAC lines. Graphic illustration created in BioRender (https://BioRender.com/ybvkq8p). (**B**) Number of tumor nodules in the lungs and the total weight of the lungs in mice injected intravenously with WT and *HS3ST1*^–/–^ F1242 and FC1245 PDAC lines. The *HS3ST1*^–/–^ lines showed a significant increase in seeding of tumors in the lung. Graph shows mean ± SEM. (**C**) Representative images of lungs from the FC1242 line experiment. Statistical analysis by 2-tailed *t* test in **B**. **P ≤ 0.01; ***P ≤ 0.001.

To examine whether HS^AT^ expression might affect experimental metastasis, which is a measure of tumor cell survival and seeding, WT and *HS3ST1*^–/–^ cells were injected into the tail vein of syngeneic WT mice, and the number of tumors seeded in the lung was determined after 2 weeks. Inactivation of *HS3ST1* in the PDAC cells resulted in increased tumor colony number and caused a significant increase in lung weight compared with WT cells in both FC1242 and FC1245 cell lines (Figure 8, A–C). Thus, HS3ST1 expression, and by inference HS^AT^, appears to affect tumor cell survival and/or seeding in this model.

### HS^AT^ sequesters endogenous AT.

**Figure 9:**
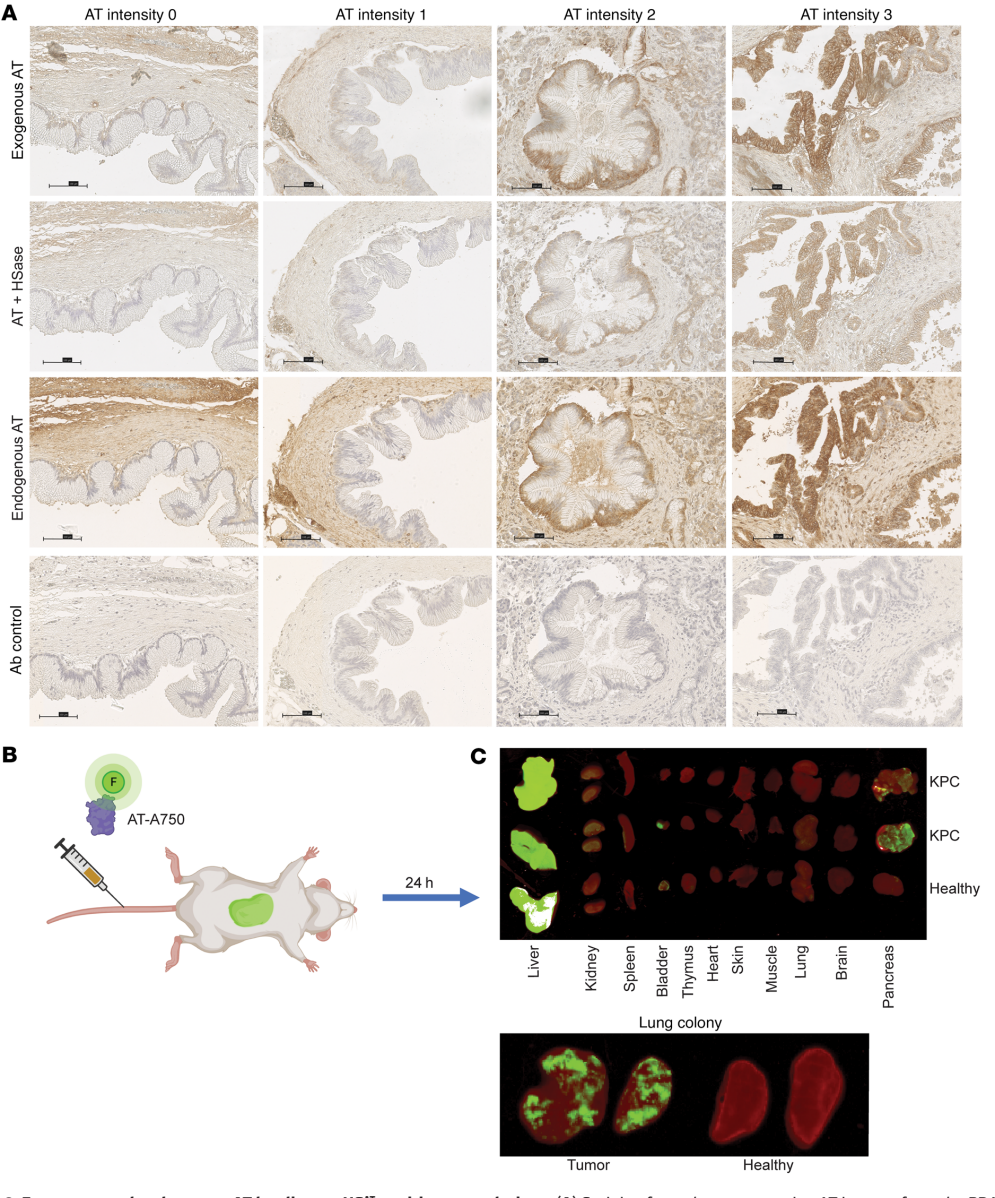
Exogenous and endogenous AT localizes to HS^AT^-positive tumor lesions. (**A**) Staining for endogenous murine AT in cores from the PDAC TMA. Staining varies but correlates to the staining intensity of HS^AT^ using exogenous human AT. The stain for endogenous AT shows more extracellular matrix staining, which may be due to the high concentration of circulating AT. (**B**) AT was labeled with Alexa750 and injected via the tail vein of mice. Created in BioRender (https://BioRender.com/9ididq0). (**C**) KPC mice were injected with labeled AT and after 24 hours, organs were harvested and scanned for fluorescent AT. AT-Alexa750 localized in pancreatic tumors, with little staining of any other organs except liver, presumably due to clearance. The lower panel shows similar staining of experimental lung metastases generated by intravenous injection of WT cells.

The presence of HS^AT^ in sections from PDAC tumors raised the question of whether endogenous host-derived AT was also bound to the tumors. Prior studies have shown that HS^AT^ is expressed in the subendothelial ECM, with very little exposed to the vascular lumen, suggesting a model in which HS^AT^ is exposed and AT is activated upon endothelial damage (14). To test whether endogenous AT is present in PDAC tumors from patients, we stained slides from the PDAC TMA for endogenous AT and compared the staining pattern to signal obtained by adding exogenous human AT. Endogenous AT was detected as diffuse ECM staining, with more intense staining in the malignant cell compartment, which correlated well with the presence and intensity of exogenous AT binding (Figure 9A). This finding suggests that endogenous AT is present in tissues expressing HS^AT^ and that not all of the binding sites are occupied.

To test whether exogenously added AT might accumulate in PDAC tumors in vivo, human AT labeled with the near-infrared fluorophore Alexa Fluor 750 (AT-A750) was injected intravenously in KPC mice with established spontaneous pancreatic tumors (Figure 9B). Organs were harvested and imaged after 24 hours, which showed a specific localization of labeled AT in the pancreatic tumors (Figure 9C). The pancreas in the healthy control did not label with AT-A750, presumably reflecting the continuous endothelium in normal pancreas that would restrict access of AT-A750 to the normal ductal epithelium. The liver also showed extensive staining by AT-A750, which can be explained by the rapid hepatic clearance of AT-protease complexes (56). Lung tumors derived by experimental metastasis also bound injected AT-750 (Figure 9C), whereas healthy lungs did not, despite HS^AT^ expression in the epithelium of this tissue (Figure 2A). In general, little localization was seen in any other tissue, corroborating reports that HS^AT^ expression in endothelium, and possibly epithelium, is not accessible to plasma AT in the healthy state (14).

### HS^AT^ regulates TF/FVIIa activity and PAR1 activation caused by thrombin.

**Figure 10:**
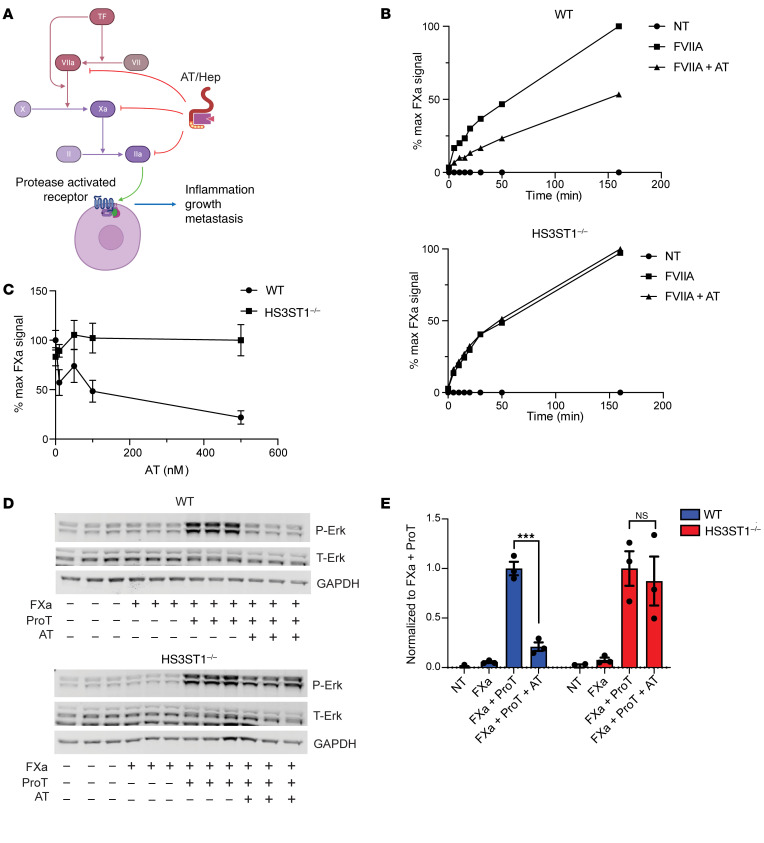
HS^AT^ expression regulates TF/FVIIa activity and thrombin-induced PAR1 activation through AT. (**A**) Schematic showing the known targets of AT in the coagulation cascade and the proposed effect of the thrombin (FIIa) on tumor function via PAR1 activation and subsequent intracellular activation of the MAPK/ERK pathway. Created in BioRender (https://BioRender.com/wqk7st3). (**B**) AT inhibits TF/FVIIa activation on WT but not on *HS3ST1*^–/–^ cells. (**C**) Titration of AT in the TF/FVIIa inhibition assay. (**D**) AT inhibits thrombin generation and PAR1 activation. (**E**) Quantification of the data in **D** after normalization to nontreated control (NT). Graph shows mean ± SEM. Statistical analysis by 2-way ANOVA with Šidák’s multiple-comparison correction. ***P ≤ 0.001; NS, not significant. Full statistical analysis shown in Supplemental Table 1. T-Erk, total Erk; ProT, Prothrombin; NT, no treatment.

Heparin binding to AT induces a conformational change, resulting in activation of AT and covalent inactivation of the serine proteases FXa and thrombin, which are involved in blood coagulation (15). Activated AT can also inactivate other serine proteases within the coagulation cascade, including FVIIa in complex with TF (57–59) (Figure 10A). The TF/FVIIa complex activates the extrinsic coagulation cascade, but the role of HS^AT^ in regulating TF/FVIIa activity in the tumor microenvironment has not been evaluated. Interestingly, multiple studies have shown that PDAC is associated with robust activation of the coagulation system through TF expression, thrombin generation, and subsequent activation of protease-activated receptor 1 (PAR-1) receptors (60–62) (Figure 10A).

To test whether HS^AT^ expression could modulate TF/FVIIa activity in tumor cells, we assayed TF/FVIIa activity on mouse PDAC cells using a modification of a previously published protocol (63). A time-dependent increase in FXa product was observed in the WT cells, indicating that TF/FVIIa generated factor Xa (Figure 10B). The addition of AT during the TF/FVIIa activation step decreased the rate of FXa product formation by about 50%. Inactivation of *HS3ST1* in the tumor cells had no effect on the activation of FXa, but completely abrogated the ability of AT to inactivate TF/FVIIa (Figure 10B), suggesting that HS^AT^ can regulate initiation of the coagulation cascade by recruiting and activating AT on the cell surface. Increasing the concentration of AT did not further inactivate TF/FVIIa in *HS3ST1*^–/–^ cells, but it had the expected effect of enhancing the inhibition of the system in WT cells (Figure 10C).

Thrombin generation through TF expression leads to PAR-1 activation and several signaling cascades including ERK1/2 phosphorylation, culminating in a pro-tumorigenic response (61). To test whether HS^AT^ regulation of the coagulation axis could affect PAR-1 activation and ERK1/2 activity, cells were grown to confluency to ensure TF expression (63), and prothrombin along with a low concentration of FXa was added to the WT and *HS3ST1*^–/–^ cells with and without AT. Addition of a low amount of FXa did not activate ERK1/2, although FXa has been shown to be able to activate PAR-1 independent of thrombin (64). Addition of prothrombin led to thrombin generation and robust activation of the cascade (Figure 10, D and E). Addition of AT inhibited this activation only in the WT cells and not in the *HS3ST1*^–/–^ cells, suggesting that HS^AT^ on the PDAC cells can recruit AT and regulate the activity of the thrombin/PAR-1 axis.

To investigate whether the ERK pathways are activated in the *HS3ST1*^–/–^ in vivo model, the transcriptome was interrogated for indicators of differential regulation by upstream transcription factors using the Ingenuity Pathway Analysis (IPA; QIAGEN) toolbox. Through running an IPA core analysis on the differentially expressed genes (adjusted *P* value < 0.05) from DESeq2, we were able to identify key signaling pathways and upstream regulators related to MAPK pathways. These were initially assessed by an overrepresentation analysis designed to capture both significance and directionality of pathway dysregulation based on predicted transcription factor activity as derived from the difference in target gene expression between *HS3ST1*^–/–^ and WT tumor cells. Results suggested potential activation of both the EMT-ome and MAPK signaling pathways in the *HS3ST1*^–/–^ tumors compared with WT tumors, based on greater predicted dysregulation of IL-17A signaling in fibroblasts, IL-1 processing, and formation of the definitive endoderm (*z* score > 1, *P* < 0.05) in *HS3ST1*^–/–^ samples. These findings motivated further analysis of the Upstream Regulator Analysis (URA) and Causal Networks (CNs) from IPA for explicit mechanisms of MAPK signaling activation that could indirectly regulate HS3ST1 expression. After ingestion of CNs targeting both HS3ST1 and MAPK signaling components, CSNK1A1, ELL, LPAR5, MAP2, NAALADL2, NFYA, SKIL, SYVN1, and YWHAG were identified as potential master regulators of both HS3ST1 and ERK components of the MAPK signaling pathway, based on shortest path from regulator to corresponding target obeying causal regulatory constraints. These networks are illustrated in Supplemental Figures 6 and 7. Taken together, these findings suggest an activation of ERK-related signaling in *HS3ST1*^–/–^ tumors, consistent with the in vitro model (Figure 10, D and E). To further illustrate this point, we performed staining for p-ERK in tumor slides from FC1245 orthotopic tumors and compared them with WT tumors, which showed a significantly increased ERK activation in *HS3ST1*^–/–^ tumors (Supplemental Figure 8).

## Discussion

The full understanding of the expression, distribution, and function of HS^AT^ has remained elusive since its original discovery (11–16). Although HS^AT^ has been isolated from several rat tissues, its cellular origin was undefined (13). HS^AT^ expression is generally thought to be rare and restricted to vascular endothelium, connective tissue mast cells, and follicular fluid (11, 12, 14–17). A key finding presented here is that HS^AT^ is ubiquitously expressed across epithelial cells in most organs (Figure 2A and Supplemental Figure 1), which corresponds with the ubiquitous expression of HS3ST1 in these cells (Figure 2B). Although the anticoagulant properties of HS^AT^ are well documented, systemic inactivation of *HS3ST1* in mice did not lead to a procoagulant phenotype, even after a challenge with thrombin (38). These findings indicate that the binding of AT and the inactivation of the coagulation cascade might not be the primary function of HS^AT^. In fact, although little effect on coagulation was shown in *HS3ST1*^–/–^ mice, the mice were susceptible to inflammatory challenges (38, 65). Thus, the functional importance of endogenous HS^AT^ may be more profound than initially suggested and possibly unrelated to anticoagulation.

We showed that HS^AT^ is predominantly expressed on the basolateral basement membrane of epithelial linings (Figure 2A and Supplemental Figure 1). Its function in this location is not clear, but by analogy to subendothelial abluminal expression of HS^AT^, it could have an anticoagulant and possibly antiinflammatory function to modulate host response to tissue injury (14, 38, 65, 66). In this model, HS^AT^ would serve a barrier function through exposure after tissue damage and subsequent AT recruitment. AT activation would then modulate the activity of serine proteases released through tissue injury. Additional studies are required to fully elucidate the function of HS^AT^ in epithelial cell barriers and to identify the potential tissue serine proteases susceptible to AT inhibition.

Of the family of 3-*O*-sulfotransferases, only HS3ST1 and HS3ST5, and possibly HS3ST4, generate the AT binding pentasaccharide sequence (17, 49). Here, we showed that HS3ST1 is expressed in epithelial cells across organs except in the prostate gland, where HS3ST5 is highly expressed (Figure 2B). The expression patterns of these isozymes correlated with HS^AT^ staining in these tissues (Figure 2A and Supplemental Figure 1). Interestingly, inactivation of *HS3ST1* led to a total reduction of HS^AT^ in the tissues as well as in the murine KPC cancer cells (Figure 6, C and E), suggesting that although multiple enzymes can generate HS^AT^, HS3ST1 is the primary enzyme responsible for HS^AT^ expression in vivo. Recent studies showed that *HS3ST1* expression in cells is regulated by ZNF263, a zinc finger transcription factor (18). However, exactly how HS3ST1 expression is controlled in PDAC remains to be elucidated.

Alterations in glycosaminoglycan expression are a hallmark of cancer development and are involved in almost every key function that promotes tumorigenesis (20–24). Pancreatic cancer is characterized by a dense stroma rich in glycosaminoglycans and marked upregulation of multiple proteoglycans (26–33). A key finding in this study was the elevated expression of HS3ST1 and the resulting HS^AT^ in the early stages of PDAC development. Interestingly, the HS^AT^ expression pattern in the tumors (Figure 4D) was similar to the staining pattern seen in the healthy pancreatic duct (Figure 3A), concentrating on the basolateral side and basement membrane of the polarized tumor cells within ductal-like tumor lesions (yellow arrowheads, Figure 4D). The correlation of HS^AT^ with ductal-like differentiation would explain the observation that HS^AT^ increases in expression across pancreatic intraepithelial neoplasia stages but declines in PDAC as the malignant tumors lose their differentiated phenotype (Figure 5, B and C). The clear HS^AT^ staining within ductal-like lesions in primary tumor specimens and the low staining in tumor lesions that fail to differentiate into ducts supports this idea (Figure 4D). Mutant KRAS is central to initial cell dysplasia (67, 68). Interestingly, GSEA revealed that *HS3ST1* inactivation in murine tumors affected KRAS signaling (Figure 7E). Furthermore, induction of mutant KRAS in a KPC mouse cell line led to a significant induction of HS^AT^ expression (Supplemental Figure 4B), suggesting that cellular changes induced by mutant KRAS lead to HS^AT^ expression.

The AT staining of the patient PDAC TMA revealed a significant correlation between HS^AT^ staining and tumor staging (Table 2). Although no significant effect was seen on primary tumor growth in the orthotopic tumor model, we did note that almost all *HS3ST1*^–/–^ tumor-bearing mice had developed ascites and peritoneal colonies, suggesting increased local spread of the tumors. Given that this orthotopic model causes rapid death due to primary tumor burden, we were unable to ascertain the impact of HS3ST1 loss on systemic metastasis. To quantify metastatic potential, we tested the WT and *HS3ST1*^–/–^ cells in a model of experimental metastatic lung seeding. Remarkably, the *HS3ST1*^–/–^ cells derived from both FC1242 and FC1245 tumors showed a dramatic increase in lung colony growth and number (Figure 8, A–C). This combined with the GSEA data (Figure 7E) suggest that HS^AT^ might modulate cellular mobility and metastasis through AT.

The function of HS^AT^ in the tumor microenvironment is unknown. We imagine that HS^AT^ normally binds AT in the tumor microenvironment and regulates the action of one or more serine proteases. Loss of AT binding would potentially increase the action of these proteases, aiding in the digestion of the surrounding ECM and in the activation of protease-sensitive receptors such as PAR-1, which in turn facilitates tumor cell migration, invasion, and eventual metastasis. Experiments are underway to examine whether similar results are observed in KPC mice lacking *HS3ST1* and to examine how host expression of *HS3ST1* affects orthotopic tumor formation. Identification of the relevant serine proteases in the system would also be of interest. The activation of PAR-1 signaling by thrombin generated in the absence of HS3ST1 and HS^AT^ could also enhance inflammation, tumor growth, and metastasis (Figure 8). It is important to note that although the HS^AT^-AT interaction is well characterized, other molecules have been suggested to preferentially interact with 3-*O*-sulfated HS (17). Thus, loss of HS^AT^ may affect multiple processes other than AT recruitment and activity.

Mice carrying homozygous deletions of *HS3ST1* did not exhibit a prothrombotic phenotype, but they did show susceptibility to inflammation when challenged (38, 65, 66). Here, we showed that knockout of HS3ST1 led to enrichment of inflammatory pathways in GSEA of dissected PDAC tumors, which is consistent with the hypothesis that HS^AT^ may play an important role in inflammation through recruitment of AT. We also showed that cellular HS^AT^ can modulate the thrombin/PAR-1 axis, which has been shown to be implicated in multiple processes, including thrombosis, cancer, and inflammation (69). Importantly, the thrombin/PAR-1 axis has been implicated in PDAC development (60–62). Although *HS3ST1*^–/–^ mice did not develop thrombotic complications, it is possible that the loss of HS^AT^ and the resulting increase in TF/FVIIa and thrombin activity could be implicated in the hypercoagulable state that is a hallmark of advanced PDAC, consistent with high TF expression in PDAC (70). Further studies should be aimed at investigating this relationship.

HS^AT^ appears to be a marker of early ductal-like dysplasia and could potentially provide a marker for such cellular transformation. Furthermore, HS^AT^ circulates in patients with tumors, although it is also present in nonmalignant disease such as pancreatitis. The latter point would limit its use as a diagnostic marker, but it could be investigated as a marker of disease progression. Since exogenously added AT targets tumors in vivo (Figure 9B), one can speculate the possible use of AT for targeting chemotherapeutics to tumors. However, it seems likely that off-target effects in other organs that express HS^AT^ would obviate this approach. Based on the concentration of injected AT in spontaneous pancreatic tumors in the KPC mouse and the experimental metastases in the lung, one might be able to engineer AT to serve as a PET probe for noninvasive imaging and to monitor chemotherapy.

We have shown that HS^AT^ is ubiquitously present in epithelial basement membranes in multiple tissues. We have further demonstrated the appearance of HS^AT^ in the early stages of pancreatic cancer. Our data suggest that expression of HS^AT^ has a tumor-suppressing function through recruitment of AT and that the loss of HS^AT^ in progression to PDAC increases inflammation and metastatic potential. Further research into HS^AT^ and its function in both healthy organs and in cancer tissue is needed.

## Methods

See Supplemental Methods for details on procedures and materials (mice, cell lines, and antibodies) used in this study.

### Sex as a biological variable.

This study included experiments with human samples and animal models. No preference was taken to one sex, and all experiments included both males and females. Human samples were completely deidentified, and information on sex was not tracked in this study. The animal studies included both female and male mice and no significant difference was seen between the sexes.

### Statistics.

Statistical analyses were performed in GraphPad Prism 10 or SPSS version 25 (IBM). All experiments were performed in triplicate and repeated at least 2 times. Normally distributed data, as determined by Shapiro-Wilk test, were analyzed statistically using unpaired 2-tailed *t* tests. Non-normally distributed data were analyzed by 2-tailed Mann-Whitney *U* test. Multiple group analysis was performed by 1-way ANOVA without post hoc correction for multiple comparisons. Survival estimates were generated using the Kaplan-Meier method and compared using log-rank tests. Multivariate Cox proportional hazards models were used to test statistical independence and significance of multiple predictors with backward selection performed using the Akaike Information Criterion. IC_50_ values, and CIs were determined using nonlinear regression using the inhibitor versus response least-squares fit algorithm. Data in figures are shown as mean ± SD or SEM, as indicated. The specific statistical tests used are listed in the figure legends. Experiments were evaluated by statistical significance; *P* values less than 0.05 were considered significant.

### Study approval.

This study was conducted at multiple institutions with approval by the respective IRBs. All animal experiments were performed according to protocols approved by the UCSD IACUC or in accordance with procedures approved by the IACUC at the Salk Institute for Biological Studies. Human tissue slides were obtained from the University of Nebraska Medical Center’s Rapid Autopsy Program for Pancreas Cancer under IRB 091-01. Patient plasma samples were collected by Moores Cancer Center Biorepository from patients who provided written informed consent under a UCSD Human Research Protections Program IRB-approved protocol (HRPP 181755).

### Data availability.

This study generated RNA-Seq data from murine PDAC cancer cell lines. The raw and processed data are uploaded to NCBI’s Gene Expression Omnibus (GEO GSE270542). Supporting data values for all graphs are available in the Supporting Data Values supplemental file.

## Author contributions

TMC, RJW, DDE, and JDE conceptualized the study. TMC, RJW, JRT, BPK, JC, LAD, JPR, IMC, TC, VP, RK, HS, KLP, SO, DRS, HJJ, GW, LPF, NB, AM, RLM, and DWD were responsible for methodology and experimental work. TR, MS, MAH, PMG, TCC, JLG, SLB, and DWD supplied reagents. AA, CH, DL, NEL, and MMF contributed essential discussions and advice. TMC and JDE conducted the main fundraising. TMC, DDE, and JDE prepared the manuscript. All authors edited the manuscript and approved it.

## Funding support

This work is the result of NIH funding, in whole or in part, and is subject to the NIH Public Access Policy. Through acceptance of this federal funding, the NIH has been given a right to make the work publicly available in PubMed Central.

- Cancer Research Coordinating Committee grant C23CR5578 to JDE.
- National Heart, Lung, and Blood Institute (NHLBI) grant HL131474 to JDE.
- Alfred Benzon Foundation to TMC.
- Dagmar Marshall Foundation to TMC.
- National Institute of General Medical Sciences (NIGMS) grant GM150736 and NHLBI grant HL167091 to RJW.
- Novo Nordisk Foundation grant NNF22OC0076899 to HJ.
- National Cancer Institute (NCI) grant CA197699 to TR.
- National Institute of General Medical Sciences (NIH) T32 grant GM007752 to LPF.
- Ruth L. Kirschstein National Research Service Award F31 CA247489 to LPF.
- NIGMS grant GM119850 to NEL.
- NCI grant CA211462 to the University of Nebraska Medical Center’s Rapid Autopsy Program.
- NCI grant U01 CA233581 to SLB.
- Novo Nordisk Foundation grant NNF22OC0073736 to RLM.
- US Department of Veterans Affairs Merit Award
- I01-BX003688-05A2 to MMF.
- NIH grant P30 CA23100 to UCSD Biorepository and Tissue Technology Shared Resources.
- NIH SIG grant S10 OD026929 to UCSD IGM Genomics Center for purchase of an Illumina NovaSeq 6000.
- Lustgarten Foundation for DDE, HS, KP, and SO.
- NIH-NCI CCSG: P30 CA01495 for DDE, HS, KP, and SO.
- Japanese Society for the Promotion of Science Oversees fellowship for SO.

## Supplementary Material

Supplemental data

Unedited blot and gel images

Supporting data values

## Figures and Tables

**Table 3:**
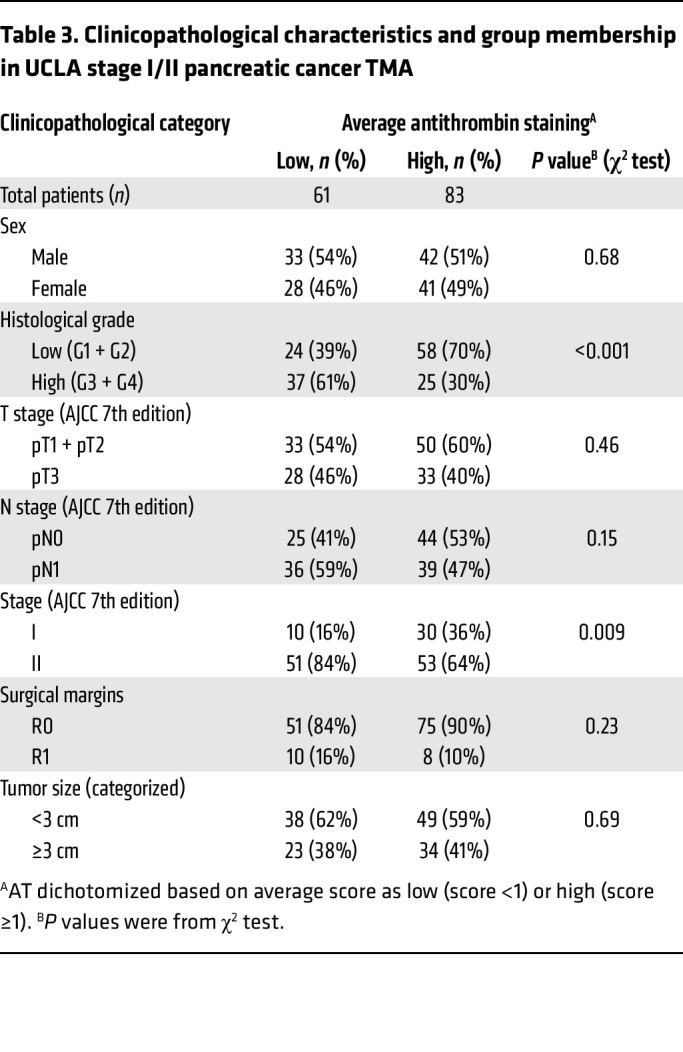
Clinicopathological characteristics and group membership in UCLA stage I/II pancreatic cancer TMA

**Table 2:**
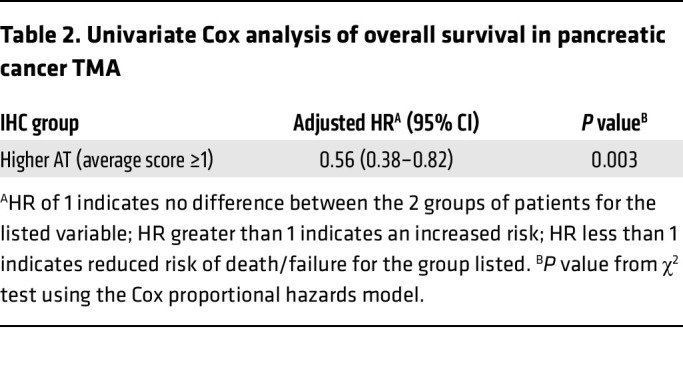
Univariate Cox analysis of overall survival in pancreatic cancer TMA

**Table 1:**
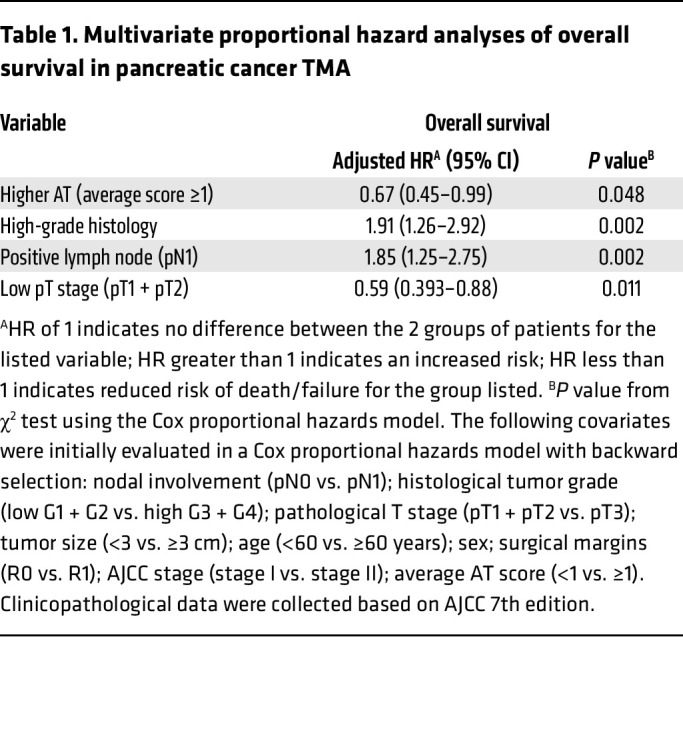
Multivariate proportional hazard analyses of overall survival in pancreatic cancer TMA

## Version 1 09/16/2025

In-Press Preview

## Version 2 11/17/2025

Electronic publication
