# Supplementary material for: Antithrombin-binding heparan sulfate is ubiquitously expressed in epithelial cells and suppresses pancreatic tumorigenesis
Source: J Clin Invest. 2025 Sep 16;135(22):e184172. doi: 10.1172/JCI184172 (PMC12618072; doi:10.1172/JCI184172)

Supplemental Methods:

Materials

Mice:

All experiments were performed according to protocols approved by the UC San Diego Institutional Animal Care and Use Committee (Protocol S99127) or in accordance with procedures approved by the IACUC at the Salk Institute for Biological Studies (Approval 18-00048). Mice were specific pathogen-free and bred and maintained in the animal care facilities at the UC San Diego or at Salk Institute for Biological Studies. C57Bl/6 mice were purchased from Jackson Laboratory. The *HS3ST1*$^{-/-}$ mouse was provided by Nick Shworak and has been previously described (1). The *LSL-Kras G12D* (*Kras*$^{G12D/+}$) mouse, B6.129S4-Kras$^{tm4Tyj/J}$ (Stock No: 008179), *p53*$^{flox/flox}$ (*p53*$^{f/f}$) mouse, and B6.129P2- Trp53$^{tm1Brn/J}$ (Stock No: 008462) mouse were purchased from The Jackson Laboratory. Dr. Chris Wright provided *p48-Cre* (*Ptf1a-Cre*) mice as previously described[34]. *LSL-R172H* mutant *p53* (p53$^{R172H/+}$), Trp53^{R172H} mice were provided by Dr. Tyler Jacks (JAX Stock No: 008183).

HS3ST1$^{-/-}$ mouse:

The line was backcrossed into the C57Bl/6 background over 10 generations. Mice were maintained as heterozygotes and in-bred to generate null mice.

LSL-Kras G12D (*Kras*$^{G12D/+}$) mouse:

All animals were maintained as mixed background. Animals had access to food and water *ad libitum* and were group-housed in ventilated cages under controlled temperature and humidity with a 12-hour light-dark cycle. Littermates were randomized into experimental groups when applicable or possible based on available mice. All mice enrolled in experimental studies were

treatment-naïve and not previously enrolled in any other experimental study. Mice were monitored bi-weekly for signs of tumor development, by both palpation and body condition.

Cell Lines:

FC1199, FC1242, and FC1245 were a gift from Dr. Dannielle Engle at the Salk Institute. The iKRAS cell line was provides by Dr. Susan L. Bellis at the University of Alabama, who received them from the original authors Ying et al (2) . Cells were maintained in DMEM, supplemented with 10% (v/v) FBS, 100 IU/mL of penicillin and 100 μg/mL of streptomycin sulfate, and the cells were grown under an atmosphere of 5% CO_2 and 95% air. Cells were passaged at ~90% confluence.

Antibodies:

Antibodies used where: anti-HS (mAb 10E4, AMSBIO, clone F58-10E40), polyclonal goat anti-serpinC1/antithrombin III antibody (R&D systems, AF1267), mouse anti-human ATIII (Santa Cruz, clone H-7), anti-Ki67 (ab15580, Abcam), 1:500 anti-CC3 (9661S, Cell Signaling Technology), 1:200 anti-pERK (4370S, Cell Signaling Technology), or 1:200 anti-Vimentin antibodies (5741S, Cell Signaling Technology), α-phospho-Erk1/2 (P-Erk, thr202/tyr204; Cell Signaling, 9101) and α-Erk1/2 (T-Erk, Cell Signaling, 9102), anti-GAPDH [D4C6R] (Cell Signaling Technology, Cat# 971665), Chicken anti-goat Alexa fluor 594 (Invitrogen, #A21468), Donkey anti-Goat IgG, Alexa Fluor™ 488 (Invitrogen, Cat# A11055), Donkey anti-goat-HRP (Santa cruz, Sc-2020), Goat-anti-mouse IgG-HRP (Biorad Labs, Cat#170-6516), Goat anti Mouse IgG, Alexa Fluor 488 (Invitrogen, A11001), Goat anti-mouse IgM, Alexa fluor 488 (Invitrogen, A21042).

Immunohistochemistry

Slide preparation from Mouse organs:

Wild-type C57Bl/6 and *HS3ST1*$^{-/-}$ mice were sacrificed and their organs were harvested for slide preparation. Full organs were washed in cold PBS and fixed for 24 hrs at 4 ^{o}C in 4% formalin. They were then washed 3 times in PBS and placed in cassettes. The cassettes were kept in 70% ethanol until processing. Paraffin imbedding and microtome cutting was performed by the UCSD mouse phenotyping core according to their workflow.

Human Tissue slides:

Slides containing deidentified human PDAC and adjacent healthy pancreas tissue were obtained from the University of Nebraska Medical Center's Rapid Autopsy Program (RAP) for Pancreas Cancer and the Normal Organ Recovery (NORs) Program (in association with Live On Nebraska), under Dr. Paul Grandgenett. These tissues were collected in compliance with IRB 091-01. The tissue microarray (TMA) has been previously described (3) and was obtained from the Dr. David Dawson (University of California Los Angeles).

Slide staining:

The slides where heated at 37 ^{o}C for 30 min and deparaffinized in xylene and rehydrated in stepwise alcohol washes (100%, 95%, 70%, and PBS). Endogenous peroxidase activity was blocked by 0.3% H_2O_2. Human AT staining and staining with mAb 10E4 (AMSBIO, clone F58-10E4) did not require antigen retrieval. Staining for endogenous AT required antigen retrieval in 0.1 M Citrate buffer at pH 6.0. Slides were blocked in 3% BSA. For staining with human AT, 20

nM purified human AT (Hyphen Biomed, PP004D) was added to the slides in 1% BSA and incubated overnight at 4 ºC). Bound AT was then detected with a polyclonal goat anti-serpinC1/antithrombin III antibody (R&D systems, AF1267) at 1:2000 dilution for 1 hr at room temperature. Bound antibody was detected using an anti-goat-HRP antibody (Santa Cruz, Sc-2020) or anti-goat-Alexa 594 antibody 1:2000 dilutions (Invitrogen, #A21468). Slides were washed in PBS between incubations. Staining for HS was performed using mouse anti-HS IgM (AMSBIO, clone F58-10E4) at 1:1000 dilution. Bound antibody was detected using an anti-mouse IgM-HRP antibody at 1:2000 dilution (Biorad Labs, Cat#170-6516). The HS specificity of the AT and 10E4 staining were assessed by pre-treating the slides with a mixture of IBEX heparin lyases (HSase) (2.5 mU/mL HSase I, 2.5 mU/mL HSase II, and 5 mU/mL HSase III) for 30 min at room temperature in PBS containing 0.5% BSA. Endogenous AT was detected using mouse anti-human ATIII (Santa Cruz, clone H-7). Bound antibody was detected using an anti-mouse IgG-HRP antibody at 1:2000 dilution (Biorad Labs, Cat#170-6516). Staining was developed using SigmaFAST DAB (Sigma, Cat. #D0426). HRP developed slides were scanned using a NanoZoomer slide scanner and images were processed in NDP.View2 (Hamamatsu). Fluorescent slides were visualized in microscopy and images were processed in ImageJ.

Scoring of PDAC patient TMA:

The tissue microarray (TMA) has been previously described (3). Three separate 1.0 mm cores for each tumor in the TMA were independently scored by a board-certified anatomic pathologist (DD) for AT activity in tumor cells as follows: 0, less than 10% membrane staining; 1, faint/barely perceptible basolateral or complete membrane staining in 10% or greater tumor cells; 2, weak basolateral or complete membrane staining in 10% or greater tumor cells; 3 moderate or

strong basolateral or complete membrane staining in 10% or greater tumor cells. The specificity of stained AT activity was confirmed by an absence of staining in an adjacent serial section pretreated with heparin lyases. For AT dichotomization, each tumor was assigned into either a low- or high-level staining group based on an average histoscore of <1 or ≥1. Survival estimates were generated using the Kaplan-Meier method and compared using log-rank tests. Multivariate Cox proportional hazards models were used to test statistical independence and significance of multiple predictors with backward selection performed using the Akaike Information Criterion. Overall survival time was measured from the date of surgery to the date of death due to any cause or last clinical follow-up. Statistical analysis was performed in SPSS version 25 (IBM).

Analysis of organ specific *HS3ST1* and *HS3ST5* mRNA expression

Data from Tabula Sapiens was retrieved (4), and for each cluster of cells corresponding to a different cell type, the pseudobulk (5) gene expression levels were calculated per gene (i.e., the trimmed mean of the UMI counts for each gene with detectable transcripts in the cluster). From this data set, the clusters for cell types corresponding to endothelial and epithelial cell types were selected, and the average expression for clusters enriched in endothelial and epithelial cells was plotted per organ.

Single-cell sequencing analysis of *HS3ST1* expression in healthy pancreas

Previously published scRNA-seq data are available under accession numbers E-MTAB-5061 and E-MTAB-5060. With the barcode-by-gene count matrix consisting of the counts of reads within each gene for each barcode, we performed cell clustering using Scanpy (v.1.6.0) (6). We normalized each barcode to a uniform read depth and extracted highly variable windows (using

thresholds: minimal mean expression >0.1 and dispersion >0.2). Then, we regressed out the total read depth for each cell, performed PCA, and extracted the top 50 principal components to calculate the nearest 30 neighbors using the cosine metric, which were subsequently used for uniform manifold approximation and projection (UMAP) dimensionality reduction with the parameters 'min_dist=0.3' and Leiden (7) (v.0.7.0) clustering with the parameters 'resolution=0.8'. We used Harmony (8) (v.0.1.0) to adjust for batch effects across experiments.

Reanalysis of bulk and single-cell RNA sequencing of human and mouse from healthy and cancerous pancreas

We examined *HS3ST1* expression in RNA sequencing (RNA-seq) data from three independent studies. We first queried *HS3ST1* expression (Fragments per kilobase transcript per million reads [FPKM], calculated from HTSeq-counts) in human pancreas across healthy and cancerous pancreas across multiple cancer stages. Data were compiled in the Pancreatic Ductal Adenocarcinoma (PDAC) cohort from The Cancer Genome Atlas (TCGA) and accessed using Genomic Data Commons (GDC). Expression at each stage was compared to healthy samples using a Wilcoxon test. We next examined human scRNA-seq (CRA001160), and cell-barcode-counts were visualized and mapped to previously computed clusters using Seurat (v.4.0.1). The data was analyzed as described above.

Finally, we examined *HS3ST1* expression a multi-cohort collection of single-cell RNA-seq from human PDAC samples (9). We used the log-ratio of median expression within each patient between type-2 and type-1 ductal PDAC (excluding samples from normal tissue). As described (9), counts were shifted log transformed to estimate expression and cells with high mitochondrial content (>25%) or extreme expression were removed. Data across cohorts were

integrated using reciprocal principal component analysis (rPCA) for batch correction and variable gene anchoring using Seurat.

Generation of *HS3ST1*$^{-/-}$ murine PDAC cell lines

Guide RNA targeting the *HS3ST1* gene (5' – GCCGACCGTCCCGCATTAGG – 3') were annealed using T4 polynucleotide kinase (New England Biolabs) and integrated into vector pSp-Cas9(BB)-2A-Puro (Addgene, a gift from Dr. Feng Zhang) using T7 ligase (New England Biolabs). The vectors were sequenced by Sanger sequencing to confirm their correct insertion. FC1199, FC1242, and FC1245 were seeded at 2 x 10^5 cells per well in 6-well plates. Transfection of the plasmids were done in serum and antibiotic free DMEM using Lipofectamine-LTX (Thermo) reagent according to the manufacturer's instructions. Clones were selected in DMEM containing puromycin (3 μg/ml). Single cell clones were achieved by staining with human AT (see FACS protocol) and selection for negative cells via cell sorting on a BD FACSAria II cell sorter (BD biosciences). Mutations in the targeted region were determined by PCR amplification and Sanger sequencing. Clones were verified using the ICE CRISPR analysis tool (Synthego).

Flow Cytometry

Cells at 50%–80% confluence were lifted with PBS containing 10 mM EDTA (GIBCO) and washed in PBS containing 0.5% BSA. The cells were seeded into a 96-well plate at 2x10^5 cells per well. A portion of the cells was treated with IBEX heparin lyase mix (2.5 mU/mL HSase I, 2.5 mU/mL HSase II, and 5 mU/mL HSase III) for 30 min at 37 °C in PBS containing 0.5% BSA. They were then incubated for 30 min at 4 °C with 100 nM human AT (Hyphen Biomed,

PP004D) or (AMSBIO, clone F58-10E4). The cells were washed twice. AT stained cells were then incubated with polyclonal goat anti-serpinC1/antithrombin III antibody (R&D systems, AF1267). The cells were washed again and incubated with anti-goat-alexa488 (Invitrogen, A11001). 10E4 stained cells were directly incubated with anti-mouse-alexa488 (Thermo Fisher; 1:1000 dilution). All incubations were done in PBS containing 0.5% BSA. The cells were washed twice and then analyzed using a FACSCanto instrument (BD Bioscience). All experiments were done a minimum of three separate times in three technical replicates. Data analysis was performed using FlowJo software and statistical analyses were done in Prism 9 (GraphPad).

HS^{AT} purification and characterization

<u>HS extraction</u>

Cells were grown to 90% confluence in 10-cm diameter plates. The cells were then washed and incubated in serum free-media for 24 hrs. The media was collected, and the cells were treated with 0.05% trypsin-EDTA (Sigma) for 10 min at 37 ^{o}C. The cells in trypsin were then centrifuged to separate trypsin buffer and cell pellet. This gave three fractions: media (secreted HS), trypsin digest (cell surface HS), and cell pellet (intracellular HS). The cell pellet was solubilized in 0.1 N NaOH for 10 min at room temperature. The solubilized pellet was then neutralized with 10 N Acetic Acid and centrifuged to remove debris. The protein concentration was determined by BCA. Pronase (Streptomyces griseus, Sigma Aldrich) was added to a final concentration of 0.4 mg/ml and Triton X-100 to a final concentration of 0.1%. The samples were then left to stir 16 hrs at 37 ^{o}C. Finally, the sample was centrifuged at 20,000 x g for 20 min to remove debris.

HS purification

HS purification was performed as previously described (10). The samples were mixed 1:10 with equilibration buffer (50 mM sodium acetate, 0.2 M NaCl, 0.1% Triton X-100, pH 6) and loaded onto a DEAE Sephacel column (GE healthcare) equilibrated with buffer. The column was washed with 50 mM sodium acetate buffer containing 0.2 M NaCl, pH 6.0, and bound GAGs were eluted with 50 mM sodium acetate buffer containing 2 M NaCl, pH 6.0. The samples were desalted on a PD10 column equilibrated in 10% ethanol (Cytiva) according to the manufacturer's instructions. The samples were lyophilized and resuspended in DNase buffer (50 mM Tris, 50 mM NaCl, 2.5 mM $MgCl_2$, 0.5 mM $CaCl_2$, pH 8.0) with 20 kU/mL bovine pancreatic deoxyribonuclease I (Sigma Aldrich) and incubated with shaking for 2 hr at 37 °C. The samples were adjusted to 50 mM Tris and 50 mM NaCl, pH 8.0, and incubated for 4 hr at 37 °C with 20 mU/mL chondroitinase ABC (Proteus vulgaris, Sigma Aldrich). To liberate the HS chains from residual peptides, the samples were β-eliminated with 0.4 M NaOH overnight at 4 °C. The HS was repurified over a DEAE column as described above.

HS structural Analysis:

Disaccharide analysis was performed largely as previously described (11). Briefly, purified cell pellet, trypsin-released, and secreted HS from WT and *HS3ST1*$^{-/-}$ FC1242 cells was lyophilized and resuspended in 50 µl of 50 mM sodium acetate buffer containing 5 mM calcium acetate, pH 6.5. Exhaustive digestion of HS by heparinase lyases (IBEX pharmaceuticals) into its disaccharide components was achieved by adding 15 mU of heparin lyase I first, then heparin lyase III, and lastly heparin lyase II with 2 hr intervals between additions while incubating at

37°C. Samples were then heat inactivated at 98 °C for 10 min and subsequently lyophilized. Disaccharide products were labelled with 2-aminoacridone (AMAC, Sigma-Aldrich) by addition of 10 μL 0.1M AMAC in 3:17 (vol/vol) acetic acid/DMSO and incubated at RT for 15 min followed by addition of 1 M $NaCNBH_3$ and incubated at 45 °C for 3 hr. Excess AMAC was removed by addition of 0.5 mL acetone followed by 20 min centrifugation at 21,000 x g, which precipitated the AMAC-tagged disaccharides. The precipitation was repeated twice. Labelled disaccharides were dissolved in 2% acetonitrile, and the disaccharide composition of the samples was determined using a Waters® Acquity® UPLC system equipped with a BEH C18 column (2.1 × 150 mm, 1.7 μm, Waters), and fluorescence at 525 nm was monitored. Ammonium acetate buffer (150 mM) containing 100 mM dibutylamine, pH 5.6, was used as mobile phase A. Mobile phase B consisted of 120 mM ammonium acetate, 80 mM dibutylamine and 20% acetonitrile, pH 5.6. The instrument was programmed to increase mobile phase B from 18.5 to 100% over 36 min at a flow rate of 0.2 mL/min for each run. For identification and quantification of disaccharides, 20 pmol AMAC-labeled disaccharide standards were injected immediately prior to the HS samples.

Anti-FXa assay:

A dilution of unfractionated heparin (Scientific Protein Laboratories) or purified HS (0.06-4 μg/ml) was mixed with human plasma AT (66 μg/ml) (Anaira) in 25 mM HEPES (N-[2-hydroxyethyl] piperazine N'- [2-ethanesulfonic acid]), 150 mM NaCl, and 0.1% BSA (pH 7.5) and 50 uL was added to each well of a 96 well plate. Human factor Xa (50 μL of 0.4 μg/ml solution; Enzyme Research Laboratories) was added to each well and incubated for 2 min at room temperature. Factor Xa specific chromogenic substrate (50 μL of 0.5 mg/ml, S-2765,

Diapharma) was added and absorbance was measured at 405 nm after 10 min. Background absorbance was subtracted prior to analysis. Data analysis and calculation of IC_{50} values was performed in GraphPad (Prism v9) and is presented as the extent of Factor Xa inhibition compared to activity with no AT present.

<u>$[^{35}S]O_4$ labeling and HS capture assay:</u>

Wild-type and *HS3ST1*$^{-/-}$ cells were seeded into 15-cm diameter plates. The cells were grown to 70% confluence and washed in F12 medium (Gibco). F12 medium containing dialyzed FBS and without antibiotics (to ensure no competing sulfate) was added and incubated for 30 min at 37 °C. 20 µCi of $[^{35}S]O_4$ (9.25–37.0 GBq/mmol; PerkinElmer Life Sciences) was then added and the cells were grown for 24 hrs. The cells were lifted with 0.05% trypsin-EDTA (Sigma) for 10 min at 37 °C. The trypsin liberated (cell surface) HS was purified as described above. ^{35}S-counts were determined on a LS 6500 Scintillation counter (Beckman Coulter). For the binding assay 5 µg human AT, bovine FGF2 (PeproTech) or BSA were incubated with 15,000 counts of $[^{35}S]$heparan sulfate for 30 min at room temperature. Samples were then added to prewashed nitrocellulose membranes on a vacuum apparatus and rapidly filtered (12). HS bound to protein stays on the membrane, whereas unbound HS flows through the filter. The membranes were then added to 2M NaCl to release bound HS. Ultima Gold XR (5 ml, PerkinElmer Life Sciences) scintillation fluid was added, and the samples were analyzed by liquid scintillation counting. The fraction of bound HS was calculated at the ratio of bound vs input material. Data analysis was performed in GraphPad (Prism v9).

Detection of HS^{AT} in patient plasma

Patient plasma samples were collected by the Moores Cancer Center Biorepository from consented patients under a University of California, San Diego Human Research Protections Program Institutional Review Board approved protocol (HRPP# 181755). Biorepository subjects provide a written consent which is maintained in the Biorepository archives.

To study if patient samples had anticoagulant heparan sulfate, HS was purified from plasma samples according to the HS purification protocol described above. Testing for HS^{AT} was done by a modified anti-FXa assay to improve sensitivity. The patient GAG was incubated with 0.6 nM of Human Factor Xa (Enzyme Research laboratory) and 40 nM of Antithrombin III (Aniara Diagnostics) with a 25 mM HEPES, 50 mM NaCl buffer (pH 7.4) for 1 hr at room temperature. The reaction was then quenched using a buffer of 0.15 M sodium acetate, 0.6% Polybrene and 1 mg/mL PEG 6000 (pH 5.0). To measure residual active Human Factor Xa, the samples were incubated with 0.75 mM of Chromogenix S-2765 (Diapharma) for 40 min at room temperature then the absorbance was read at 405 nm. A standard heparin curve using medical grade heparin (McKesson) was ran alongside the patient samples with the same conditions and read at the same time. Samples that had low OD values implied that the purified GAG can activate AT and therefore inactivate the FXa. To determine if the FXa inhibition was dependent on heparan sulfate, the patient GAG was treated with 0.4 mU of IBEX heparin lyases I, II, and III for 30 min at 37°C, then used in the assay as described.

***In Vivo* Tumor models**

Orthotopic grafted Models

All animal experiments were conducted in accordance with procedures approved by the IACUC at the Salk Institute for Biological Studies. For the orthotopic engraftment of mouse PDAC cells,

mice were anesthetized using isoflurane. An incision was made in the left abdominal side. PDAC cells (approximately $1x10^3$ cells/mouse) were prepared from fresh cultures. Cells were washed with ice-cold PBS and resuspended in Matrigel (Corning) diluted 1:1 with cold DMEM/F-12 containing 5% FBS. The 50 µl cell suspension was injected into the tail region of the pancreas using insulin syringes (29 guage). Successful injection was verified by the appearance of a fluid bubble without signs of intraperitoneal leakage. The abdominal wall was sutured with absorbable Vicryl suture (Ethicon), and the skin was closed with wound clips and tissue glue (Vetbond). Tumor growth was measured using the Vevo 3100 ultrasound system (Fujifilm). Mice were euthanized once tumors reached a minimum width and length of 6x6 mm. Blood was collected in EDTA coated tubes via cardiac puncture. Plasma was isolated from blood by centrifugation at >10,000 rpm at 4 ^{o}C, and snap frozen using liquid nitrogen. Tumors were harvested, washed with PBS, and any residual normal pancreas was removed. Tumors were sectioned into multiple pieces for RNA isolation (TRIzol) and histology (10% Formalin).

Experimental metastasis – intravenous lung seeding model

FC1242 and FC1245 WT and *HS3ST1*$^{-/-}$ cells were grown to 70% confluence and detached in EDTA. The cells were resuspended in PBS without calcium and magnesium and were mixed rigorously to ensure single cell suspension, as visualized by microscopy. Cells were counted three times, and the average was used to determine the concentration. Cells (50,000) were then injected intravenously through the tail vein into wild-type C57Bl/6 mice. Mice were checked and weighed bi-daily to ensure their health. The mice were sacrificed 2 weeks post injection, and the lungs were removed and weighed. Tumor colonies were counted by visual inspection using a dissection microscope. Data analysis was performed in GraphPad Prism v9.

In vivo AT localization studies

Human AT (Hyphen Biomed, PP004D) was labelled with alexa750 according to the manufacturer's instructions. Briefly, AT was mixed with 3-fold molar excess Alexa750-NHS (Thermo) and incubated 1 hr at room temperature. The reaction was quenched in 1 M glycine for 10 min. Labelled AT was then purified by buffer exchange using a 7 kDa cutoff Zeba column (Thermo) according to the manufacturer's instructions. The labeled AT in PBS (10 μg) was then injected intravenously through the tail vein into tumor bearing mice. The mice were sacrificed 24 hr post injection, and the organs were harvested and scanned for fluorescence using a LI-COR Odyssey fluorescence reader.

Staining of mouse tumors sections

Tissues were fixed in 10% neutral-buffered formalin for 24 hr, washed with 70% ethanol, embedded in paraffin, and sectioned (5-μm). For immunohistochemistry (IHC), heat-induced antigen retrieval was performed in 10 mM sodium citrate buffer (pH 6.0) or 1 mM EDTA (pH 8.0) in a pressure cooker for 20 min after dewaxing. Endogenous peroxidases were quenched with 3% H_2O_2 in Tris-buffered saline (TBS) for 20 min at room temperature. Tissue sections were blocked with 10% goat and 2.5% horse sera and incubated with primary antibody overnight at 4 °C. The following primary antibodies were diluted with blocking buffer: 1:200 anti-Ki67 (ab15580, Abcam), 1:500 anti-CC3 (9661S, Cell Signaling Technology), 1:200 anti-pERK (4370S, Cell Signaling Technology), or 1:200 anti-Vimentin antibodies (5741S, Cell Signaling Technology). The sections were incubated with primary anitibody for 1 hr at room temperature, followed by washing with TBS with 1% Tween 20 (TBST) and incubation with secondary

antibody (ImmPRESS HRP IgG Polymer Detection Kit, Vector Laboratories) for 30 min at room temperature followed by washing with TBST. The signals were developed with diaminobenzidine substrate (ImmPACT DAB HRP Substrate kit, Vector Laboratories). Slides were counterstained with hematoxylin, dehydrated, and cover slipped following standard protocol.

Quantification of IHC staining

IHC slides were scanned and processed in QuPath ver 0.4.3. Scanned slides were opened as brightfield (H-DAB) in QuPath, and the intensity of the DAB stain and the hematoxylin counterstain were adjusted using Estimate Stain Vectors Function. Cells were annotated based on their morphologies to allow QuPath to classify each cell type automatically. Three to five tumor regions were randomly selected per slide and positive cells were detected using the Cell Detection function after creating full image annotation.

RNA isolation, purification, and RNA-Seq.

Tumor samples were incubated with TRIzol (ThermoFisher) and homogenized using the TissueLyser II system (Qiagen). Samples were centrifuged at >10,000 rpm to remove cell debris. Samples were incubated with 0.2 mL chloroform per 1 mL TRIzol for 2-3 min at room temperature. To separate the aqueous phase, samples were centrifuged at >10,000 rpm for 18 min at 4 ^{o}C. The aqueous phase was separated into a new tube and an equal part of 70% Ethanol was added. RNA was isolation using the RNEasy kit (Qiagen) as described by the manufacturer with one extra Buffer RPE wash. RNA integrity was verified on a Tapestation (Agilent). Library

generation and sequencing was performed per standard protocol utilizing an Illumina NovaSeq 6000 by the UC San Diego IGM Genomics Center.

Tumor RNA-Seq analysis (Metascape and GSEA).

Quality control, RNA library preparations, and sequencing reactions of the extracted RNA from PDAC tumors were performed at the UC San Diego IGM Genomics Core. FASTQ files were aligned against Mus musculus M27 GENCODE genomic assembly using STAR and quantified using RSEM. Code for these processes can be found at https://github.com/jkccoker/Murine_PDAC_RNAseq. Low counts (<1 CPM) were filtered out using ComBat and differential expression analysis was carried out using DESeq2 in DEBrowser (v1.28.0a) (13). Genes with an adjusted p-value <0.05 were assigned as differentially expressed. Gene ontology analysis and Gene Set Enrichment Analysis (GSEA) of differentially expressed genes were carried out using Metascape (14) and GSEA (v4.3.3) (15, 16), respectively. The raw and processed data are uploaded to NCBI GEO database (Accession #GSE270542).

TF/FVIIa experiment

The ability of cell surface HS^{AT} to bind and activate AT to inhibit cell surface TF/FVIIa activity was measured by its ability to inhibit the generation of FXa, according to a modification of a previously published method (17). Wild-type and *HS3ST1*$^{-/-}$ cells were grown to confluence to achieve robust TF expression (17). The cells were then washed twice in Buffer A (10 mM HEPES, 150 mM NaCl, 4 mM KCl, 11 mM glucose, pH 7.45). Human FVIIa (Innovative Research) in buffer A was added to a final concentration of 10 nM and incubated for 1 hr. The cells were then washed 6 times in ice-cold buffer B (Buffer A plus 5 mM $CaCl_2$, 1 mg/ml BSA)

to remove unbound FVIIa. The cells were then treated with human AT (10 - 500 nM) for 15 min in buffer B. The monolayer was then overlaid with 0.5 ml FX (175 nM, Innovative Research) in buffer B. An aliquot (50 µL) was taken at different time intervals and added to 0.2 mL stopping buffer (50 mM Tris, 150 mM NaCl, 5 mM EDTA, 1 mg/ml BSA, pH 7.5). An aliquot (50 µL) was mixed with 50 µL FXa specific substrate (1.25 ml/ml, S-2765, Diapharma) and absorbance was measured at 405 nM. Data analysis was performed in GraphPad Prism (v9).

ERK1/2 western blotting and thrombin activation assay

Wild-type and *HS3ST1*$^{-/-}$ cells were seeded in 6-well plates in triplicates. The cells were grown to 70% confluency and serum starved for 24 hrs. The media was removed so that 1 mL remained in the well. Human AT (100 nM) was added to the cells and incubated 10 min at room temperature. Purified human FXa (0.1 µg, Innovative Research) was then added and incubated for 2 min. The reaction was then started by the addition of 1 µg human purified prothrombin (Innovative Research). Following a 10 min incubation, cells were quickly washed in cold PBS and then lysed in RIPA lysis buffer containing phosphatase and protease inhibitor cocktails (Roche). The samples were cleared by centrifugation and the protein concentration was determined by BCA. The samples were balanced to ensure equal protein loading. The samples were then analyzed by SDS-PAGE and Western blotted with α-phospho-Erk1/2 (P-Erk, thr202/tyr204; Cell Signaling, 9101) and α-Erk1/2 (T-Erk, Cell Signaling, 9102). α-GAPDH (D4C6R, Cell Signaling Technology, Cat# 971665) was used as the loading control. T-Erk was used to account for total ERK expression. Band intensity were determined in ImageJ. Data analysis was performed in GraphPad Prism (v9).

Ingenuity Pathway Analysis

QIAGEN IPA (QIAGEN Inc) software (18) was employed to interrogate the log2FCs in gene expression from comparison of wild-type and *HS3ST1*$^{-/-}$ samples for indicators of differential regulation by upstream transcription factors. All differentially expressed genes (adjusted p-value <0.05) from the DESeq2 analysis were used to run an IPA core analysis stipulating a background gene set composed of genes with base mean expression across wild-type and *HS3ST1*$^{-/-}$ samples within the base mean bounds of all differentially expressed genes. A subsequent Over Representation Analysis (ORA) designed to assess the significance of pathway activation directionality was performed on analysis ready molecules resulting fom the core analysis, using the pre-existing set of canonical pathway-associated gene lists internally maintained by IPA. For each canonical pathway (n = 1143), both a Z-score determining the agreement in activation status of each pathway in relation to a canonical pathway pattern as established by a Qiagen curator, and a p-value (right-tailed Fisher's exact test) determining significance of overlap between analysis ready molecules and the entire gene background encompassing all canonical pathways were calculated. Observing that pathways associated with genes participating in the epithelial-mesenchymal transition (EMT) were upregulated in *HS3ST1*$^{-/-}$ samples, an Upstream Regulator Analysis (URA) resulting from the core analysis was leveraged to interrogate EMT-ome interaction with the MAPK signaling pathway. This comparison was performed by extracting all transcription factors (TFs) and targets meeting the following criteria: (i) an absolute z-score > 2 indicating predicted activation or inhibition of the TF dependent on score sign, (ii) an overlap p-value < 0.05 indicating significant representation of the TFs known set of target molecules, (iii) agreement between the predicted activation status of the upstream regulator as informed by individual and consensus gene expression, and (iv) at least one target of

the TFs, or the TF itself belonging to the set of EMT-ome genes as retrieved from EMTome (19). Causal Network(CNs) resulting from the core analysis were leveraged to interrogate the potential relationship between HS3ST1 and predicted master regulators of the MAPK signaling pathway. This comparison was performed similarly to the interrogation of the URA for EMT-ome interaction with MAPK signaling. The resultant network was curated as follows continuing from step (iii) of the URA post-processing by (iv) assigning the labels of (a) master regulator, (b) intermediate regulator, (c) master and intermediate regulator, (d) master regulator with *HS3ST1* as downstream target, (e) master and intermediate regulator with *HS3ST1* as downstream target to the MAPK signaling pathway genes extracted from the IPA canonical pathway gene list and captured in CNs, (v) assigning the same labels to TFs from CNs that target members of the MAPK signaling pathway but are not members, and(vi) merging CNs based on shared label and gene symbol.

Supplemental Tables:

Supplemental table 1. Sidak's multiple comparison from Fig 10E.

Šídák's multiple comparisons test	Mean diff.	95.00% CI of diff.	Adjusted P Value	Summary
**WT**				
NT vs. FXa	-0.05498	-0.5282 to 0.4183	0.9996	ns
NT vs. FXa + ProT	-1	-1.473 to -0.5268	<0.0001	****
NT vs. FXa + ProT + AT	-0.211	-0.6843 to 0.2622	0.7378	ns
FXa vs. FXa + ProT	-0.945	-1.418 to -0.4718	0.0001	***
FXa vs. FXa + ProT + AT	-0.156	-0.6293 to 0.3172	0.9155	ns
FXa + ProT vs. FXa + ProT + AT	0.789	0.3157 to 1.262	0.0008	***
**HS3ST1**				
NT vs. FXa	-0.07753	-0.5508 to 0.3957	0.9974	ns
NT vs. FXa + ProT	-1	-1.473 to -0.5268	<0.0001	****
NT vs. FXa + ProT + AT	-0.8736	-1.347 to -0.4003	0.0003	***
FXa vs. FXa + ProT	-0.9225	-1.396 to -0.4492	0.0001	***
FXa vs. FXa + ProT + AT	-0.796	-1.269 to -0.3228	0.0007	***
FXa + ProT vs. FXa + ProT + AT	0.1264	-0.3468 to 0.5997	0.9674	ns

Supplemental Figure Legends

Supplemental Figure 1. HS^{AT} is expressed by epithelial cells across different organs. (A) AT staining of HS^{AT} in FFPE tissue section from various organs isolated from wild-type (WT) and *HS3ST1*$^{-/-}$ mice. mAB 10E4 is used to stain for general HS. HS^{AT} staining occurs on epithelial cells across all tested organs and is dependent on expression of *HS3ST1*. Scale bar set at 100 μm. (B) AT staining of HS^{AT} and general HS (10E4) in FFPE tissue section from various organs isolated from wild-type (WT) mice with and without pretreatment with HSase. HSase treatment abrogates binding of both AT and mAb 10E4. The images without HSase treatment also appear in Fig. 2A, and are included here again to allow for direct comparison. Scale bar set at 100 μm.

Supplemental Figure 2. (A) AT staining of fixed KPC cells grown in monolayer culture, with and without heparin lyase (HSase) treatment. Bound AT, green; DAPI stained nuclei blue. **(**B) DNA sequencing of HS3ST1$^{-/-}$ mutants. Wild-type DNA is depicted above with indicated PAM and sgRNA target sequence. Sequencing of both FC1242 and FC1245 mutants showed efficient *HS3ST1* editing inducing frameshift. Analysis performed using the Synthego ICE tool.

Supplemental Figure 3. HPLC chromatograms showing disaccharide composition of AMAC-labelled heparin lyase treated HS from wild-type and *HS3ST1*$^{-/-}$ FC1242 cells. Disaccharide standards shown on top were run prior to samples for identification and quantification of disaccharides in samples. The dotted lines indicate elution times for the disaccharides illustrated above the chromatograms. The ΔUA-GlcNAc6S disaccharide elutes at the same time as a fragment of the heparinases, and cannot be reliably quantified here.

Supplemental Figure 4. HS^{AT} circulates in plasma from patients with pancreatic cancer and pancreatitis. HS was purified from patient plasma and the presence of HS^{AT} was detected using the anti-FXa assay. The graph shows the ability of a given HS sample to activate AT and therefore inhibit thrombin generation and colorimetric development (OD450). Pretreatment of the sample with HSase abrogates AT activation and thrombin inhibition. Statistical analysis by Sidaks multiple comparison test. ****, $P \leq 0.0001$; ns, not significant.

Supplemental Figure 5. (A) Hematoxylin/eosin (H&E) and AT staining of FFPE tissue sections derived from tumors. Genetic inactivation of *HS3ST1* diminished AT staining. Some focal HS^{AT} remains (red arrows), suggesting some infiltrating cells from the host expressed HS^{AT} in the tumor microenvironment. (B) Staining of FFPE tissue sections derived from the FC1245 tumors for Vimentin. Hematoxylin/eosin (H&E) and Vimentin stain is shown. Quantifcation of staining intensity is illustrated in the graph. Colored dots represent mean staining intensity for each tumor. White dots represent staining intensity per image field. (C) Binding of AT to iKRAS cells after 72 hrs of doxycycline exposure to induce *KRAS* expression. Right panel shows expression of the *KRAS* construct after doxycycline exposure. Statistical analysis by Mann-Whitney test in B. *, $P \leq 0.05$.

Supplemental Figure 6. Manual reconstruction of MAPK regulatory network through ingestion of causal networks from IPA. Predicted activation status (abs(z-score) > 2) of TF in *HS3ST1*$^{-/-}$ as compared to WT is represented by node outline and edge, where red indicates activation and blue inhibition of the TF in the KO relative to the wildtype (WT). Known relationship as established from IPA literature base is represented by edge shape, where arrow indicates known upregulation and bar downregulation of the downstream target by the upstream TF. Actual log2FC in expression between conditions is represented by node fill, where orange indicates higher and green lower expression of the TF or target in the KO relative to wildtype. Grey node outlines are reserved for target genes with no predicted downstream regulatory activity. White node fill is reserved for TF families or complexes consisting of multiple members with predicted activation status' in aggregate that support activation or inhibition. Note that regulator activity and expression may not agree, as regulator activity is predicted from expression directionality of known target genes.

Supplemental Figure 7. Overlay of regulatory network on MAPK signaling pathway. Log2FC in expression between conditions is represented by node fill, where red indicates higher and green lower expression of pathway member in *HS3ST1*$^{-/-}$ as compared to the wildtype. Highlights on pathway member(s) represent differential expression between KO and wildtype (WT, yellow), and participation as master/intermediate regulator (purple) or target (pink) in regulatory network. Fill of pathway nodes representing multiple pathway members is mean log2FC.

Supplemental Figure 8. Staining of FFPE tissue sections derived from the FC1245 tumors for p-ERK. pERK stain is shown. Quantifcation of staining intensity is illustrated in the graph. Colored dots represent mean staining intensity for each tumor. White dots represent staining intensity per image field. Statistics by Mann-Whitney test. *, P ≤ 0.05.

Supplemental References:

1. Shworak NW, et al. Mice deficient in heparan sulfate 3-O-sulfotransferase-1: normal hemostasis with unexpected perinatal phenotypes. *Glycoconjugate journal.* 2002;19(4-5):355-61.
2. Ying H, et al. Oncogenic Kras maintains pancreatic tumors through regulation of anabolic glucose metabolism. *Cell.* 2012;149(3):656-70.
3. Manuyakorn A, et al. Cellular histone modification patterns predict prognosis and treatment response in resectable pancreatic adenocarcinoma: results from RTOG 9704. *Journal of clinical oncology : official journal of the American Society of Clinical Oncology.* 2010;28(8):1358-65.
4. Tabula Sapiens C, et al. The Tabula Sapiens: A multiple-organ, single-cell transcriptomic atlas of humans. *Science.* 2022;376(6594):eabl4896.
5. Dworkin LA, et al. Applying transcriptomics to studyglycosylation at the cell type level. *iScience.* 2022;25(6):104419.
6. Wolf FA, et al. SCANPY: large-scale single-cell gene expression data analysis. *Genome Biol.* 2018;19(1):15.
7. Traag VA, et al. From Louvain to Leiden: guaranteeing well-connected communities. *Scientific reports.* 2019;9(1):5233.
8. Korsunsky I, et al. Fast, sensitive and accurate integration of single-cell data with Harmony. *Nature methods.* 2019;16(12):1289-96.
9. Chijimatsu R, et al. Establishment of a reference single-cell RNA sequencing dataset for human pancreatic adenocarcinoma. *iScience.* 2022;25(8):104659.
10. Esko JD. Special considerations for proteoglycans and glycosaminoglycans and their purification. *Current protocols in molecular biology.* 1993;22(1):17.2. 1-.2. 9.
11. Karlsson R, et al. Dissecting structure-function of 3-O-sulfated heparin and engineered heparan sulfates. *Science advances.* 2021;7(52):eabl6026.
12. Kreuger J, et al. Nitrocellulose filter binding to assess binding of glycosaminoglycans to proteins. *Methods Enzymol.* 2003;363:327-39.
13. Kucukural A, et al. DEBrowser: interactive differential expression analysis and visualization tool for count data. *BMC genomics.* 2019;20(1):6.
14. Zhou Y, et al. Metascape provides a biologist-oriented resource for the analysis of systems-level datasets. *Nat Commun.* 2019;10(1):1523.
15. Subramanian A, et al. Gene set enrichment analysis: a knowledge-based approach for interpreting genome-wide expression profiles. *Proceedings of the National Academy of Sciences of the United States of America.* 2005;102(43):15545-50.

16. Mootha VK, et al. PGC-1alpha-responsive genes involved in oxidative phosphorylation are coordinately downregulated in human diabetes. *Nat Genet.* 2003;34(3):267-73.
17. Le DT, et al. Relations between factor VIIa binding and expression of factor VIIa/tissue factor catalytic activity on cell surfaces. *The Journal of biological chemistry.* 1992;267(22):15447-54.
18. Kramer A, et al. Causal analysis approaches in Ingenuity Pathway Analysis. *Bioinformatics.* 2014;30(4):523-30.
19. Vasaikar SV, et al. EMTome: a resource for pan-cancer analysis of epithelial-mesenchymal transition genes and signatures. *British journal of cancer.* 2021;124(1):259-69.

Supplemental Fig. 1

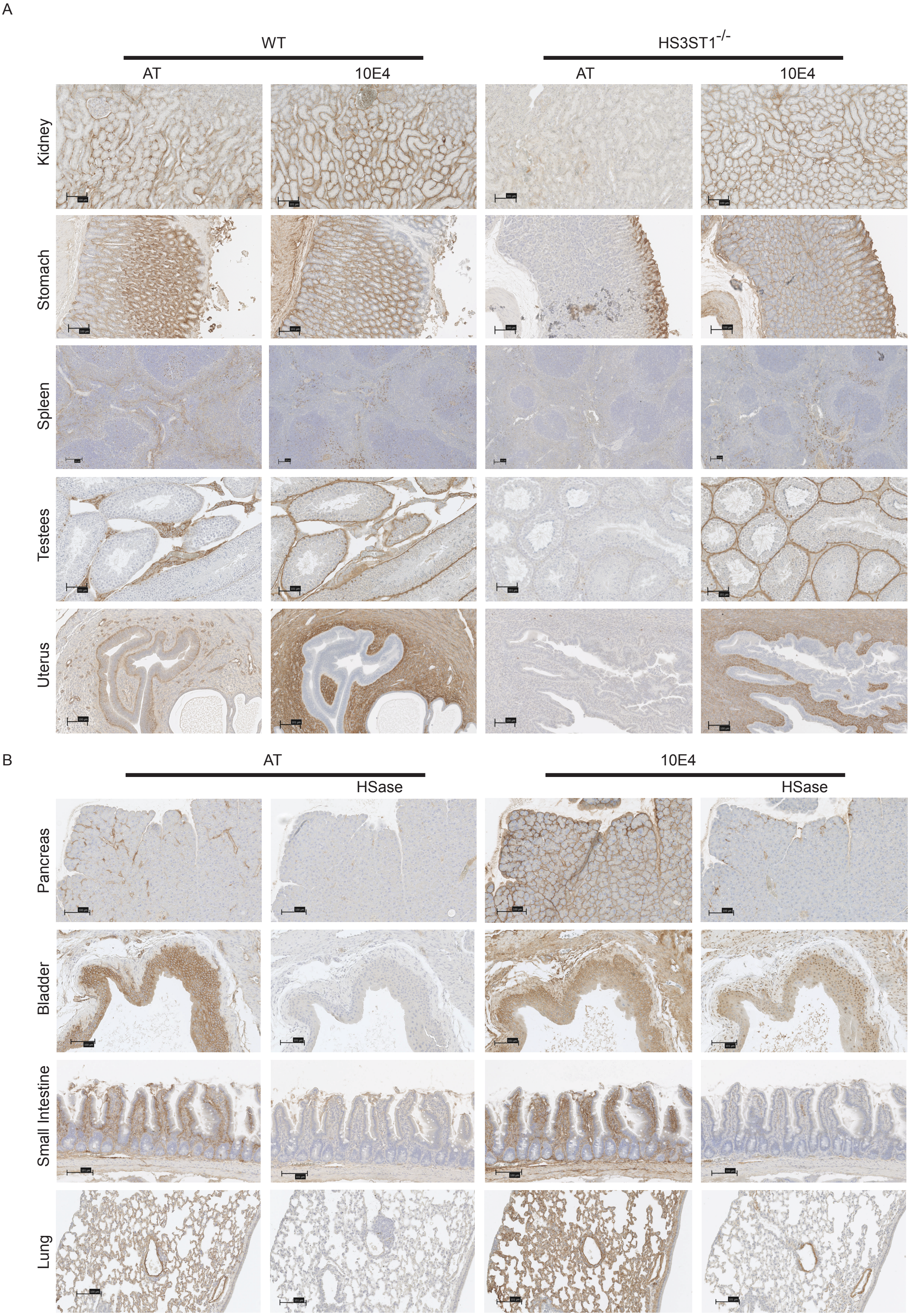

Supplemental Fig. 2

A

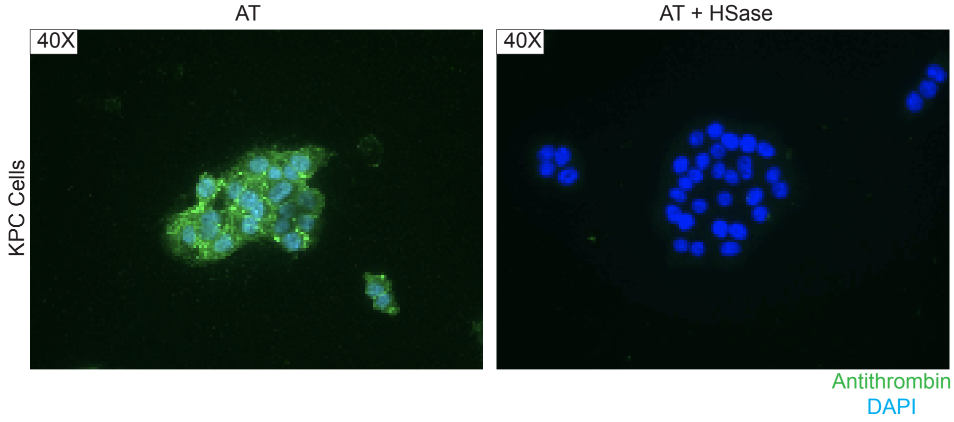

B

PAM sgRNA

HS3ST1 WT ATCCACCCATTGAGGACCT**CCT****AATGCGGGACGGTCGGC**TG

FC1242 *HS3ST1*$^{-/-}$

ATCCACCCATTGAGGACCTCCTNNTGCGGGACGGTCGGCTG (+2, fs)

ATCCACCCATTGAGGACCTCCTNATGCGGGACGGTCGGCTG (+1, fs)

ATCCACCCATTGAGGACCTC - - AATGCGGGACGGTCGGCTG (-2, fs)

FC1245 *HS3ST1*$^{-/-}$

ATCCACCCATTGAGG- - - - - - - - - - - - - GGACGGTCGGCTG (-13, fs)

ATCCACCCATTGA - - - - - - - - - - - - - - GGGACGGTCGGCTG (-14, fs)

Supplemental Fig. 3

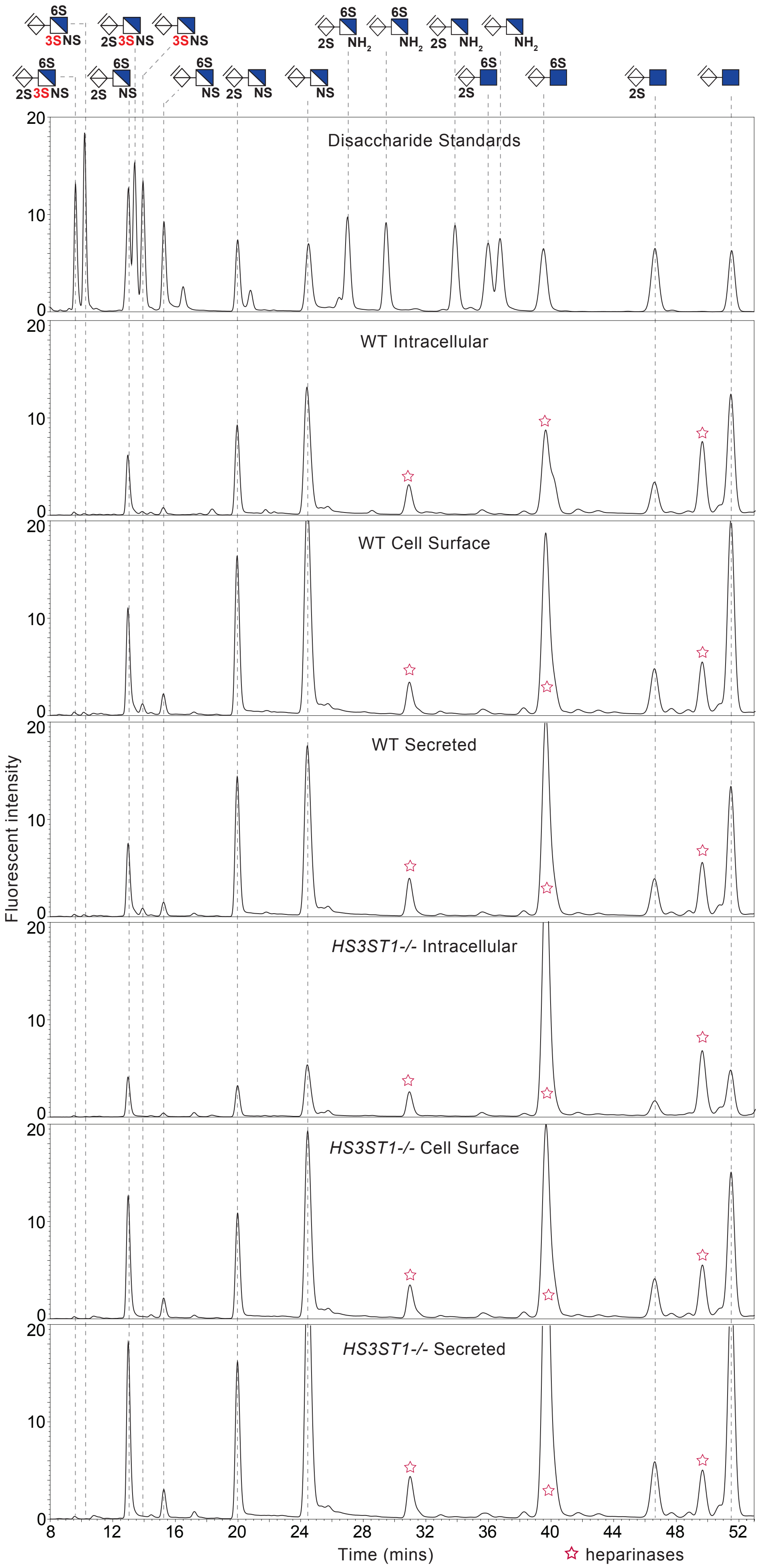

Supplemental Fig 4.

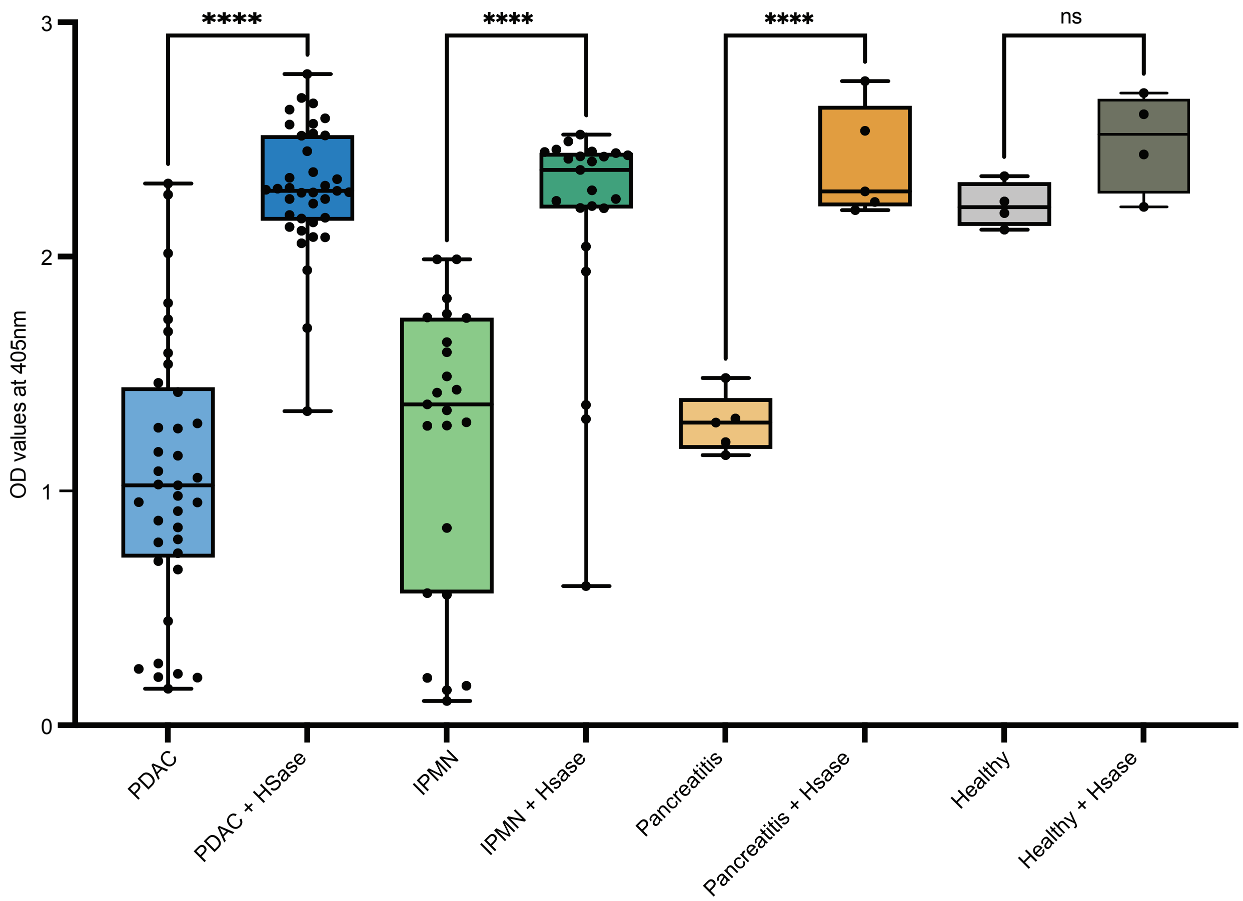

Supplemental Fig. 5

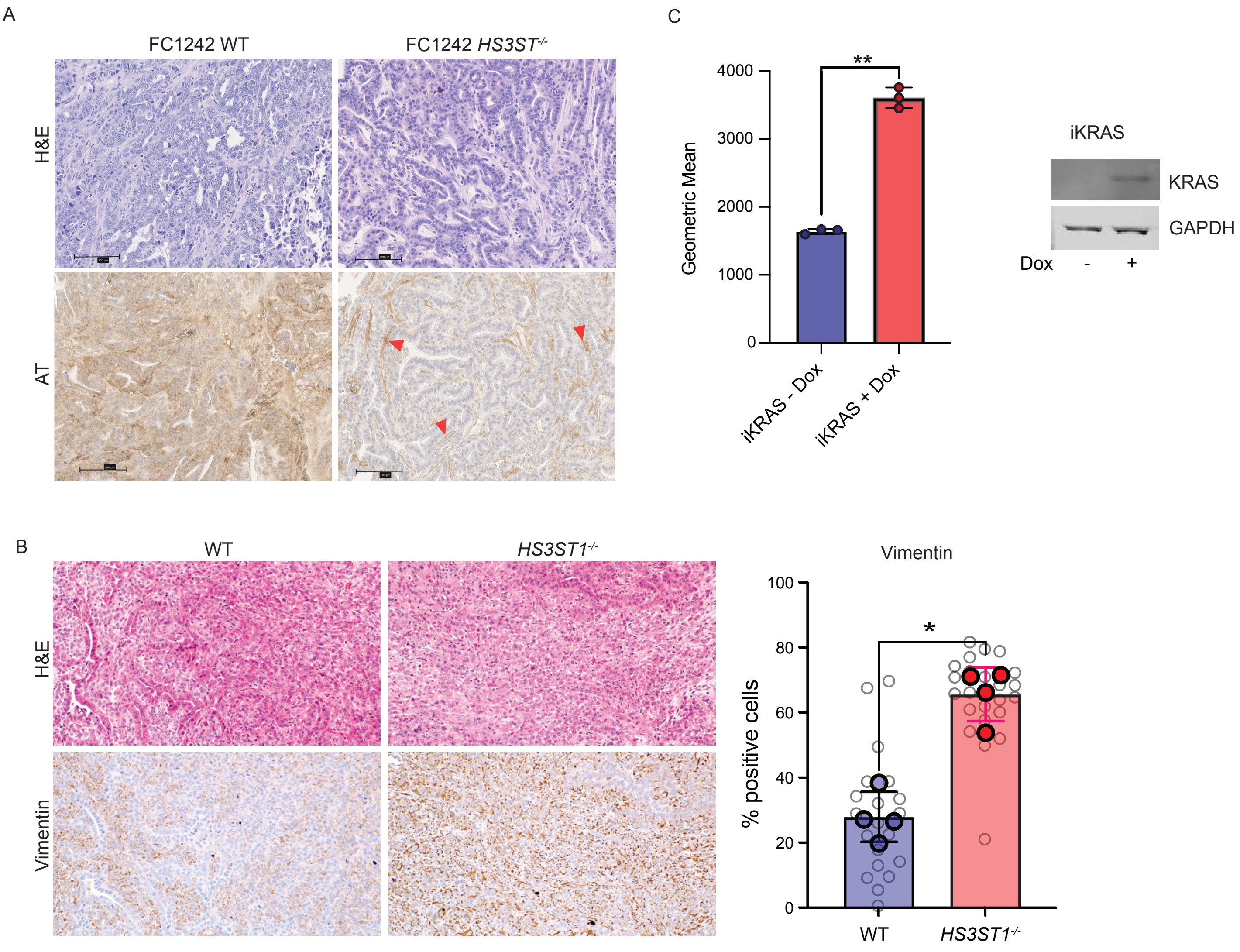

Supplemental Fig. 6

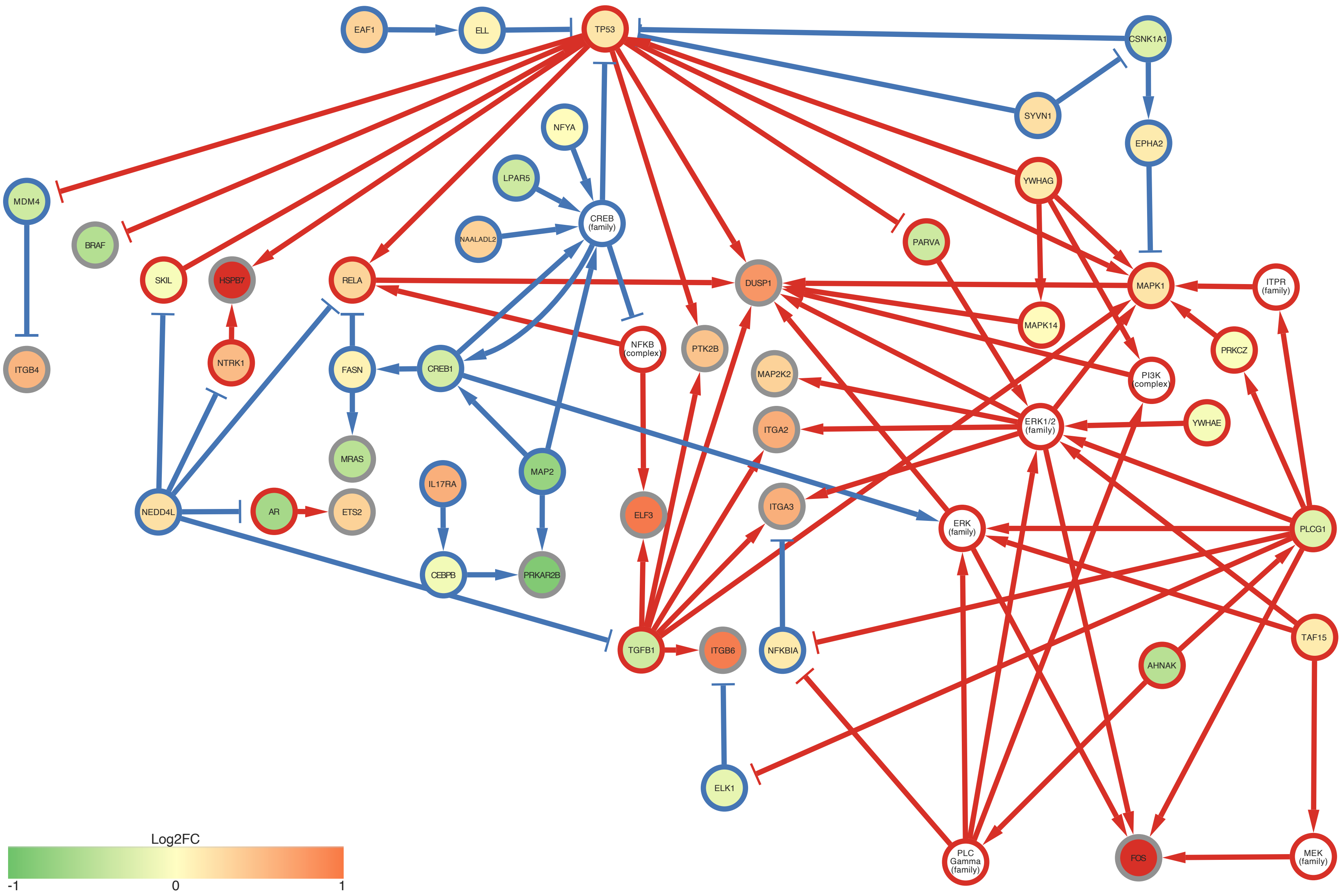

Supplemental Fig. 7

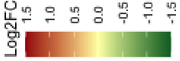

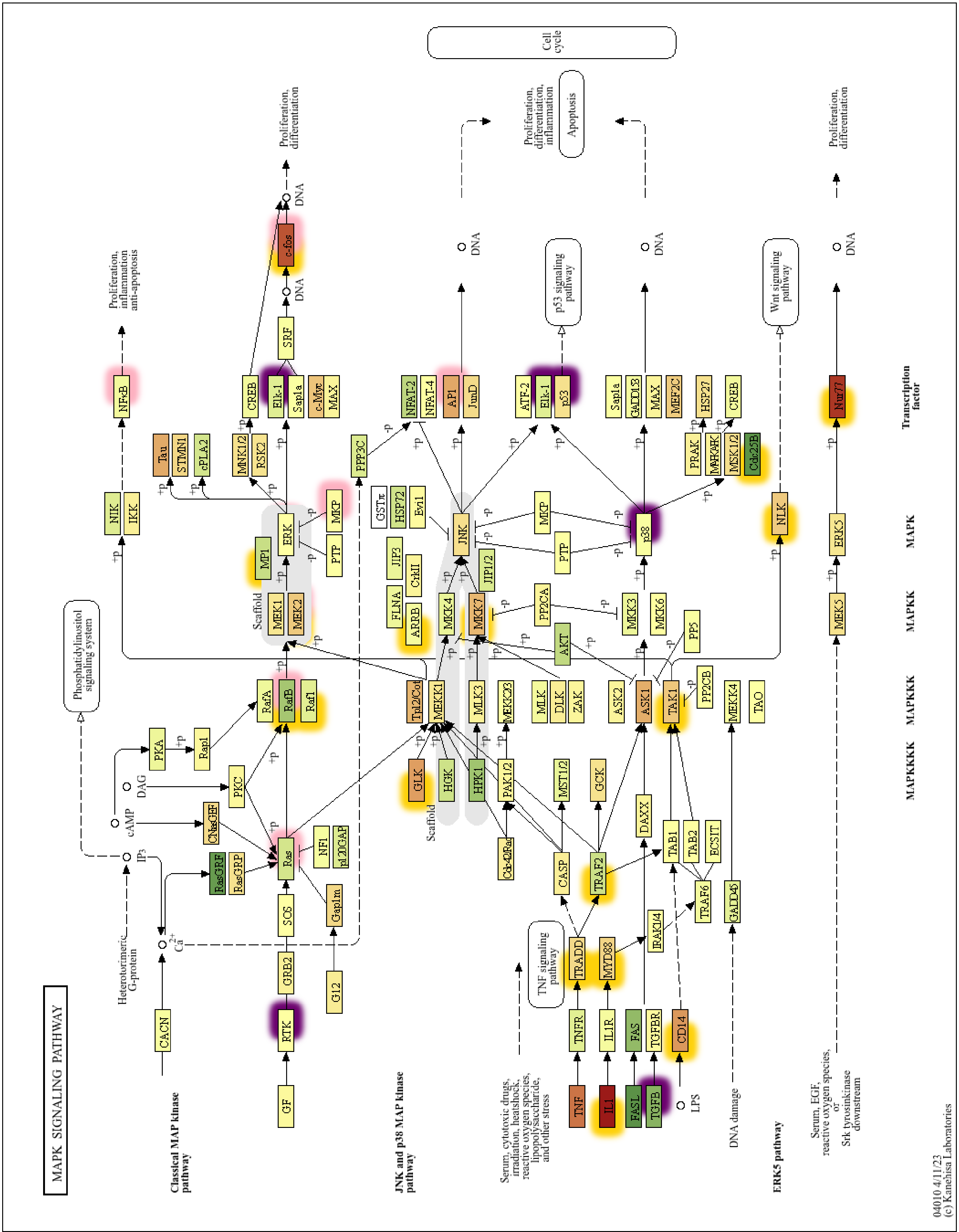

Supplemental Fig. 8

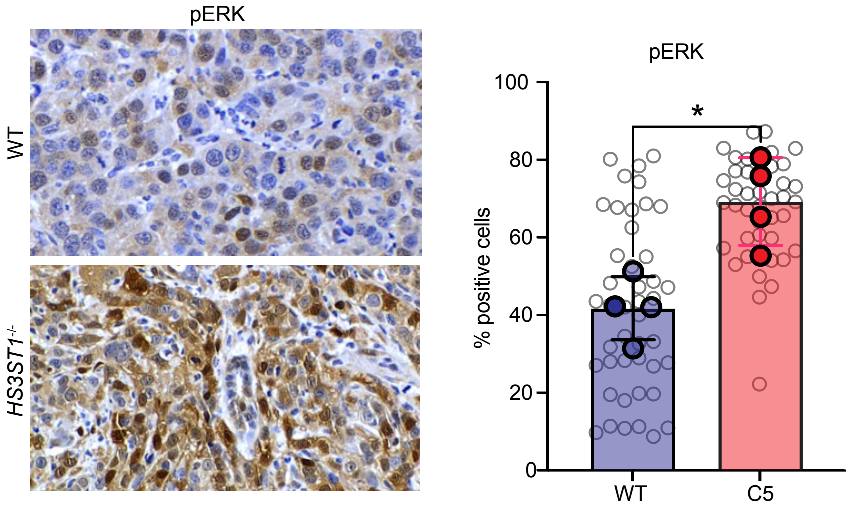

Supplement: Supplemental data [file jci-135-184172-s179.pdf]
