# Supplementary material for: Antithrombin-binding heparan sulfate is ubiquitously expressed in epithelial cells and suppresses pancreatic tumorigenesis
Source: J Clin Invest. 2025 Sep 16;135(22):e184172. doi: 10.1172/JCI184172 (PMC12618072; doi:10.1172/JCI184172)
Supplement: Unedited blot and gel images [file jci-135-184172-s180.pdf]

Full unedited blot for WT in **Figure 10D**

WT cells

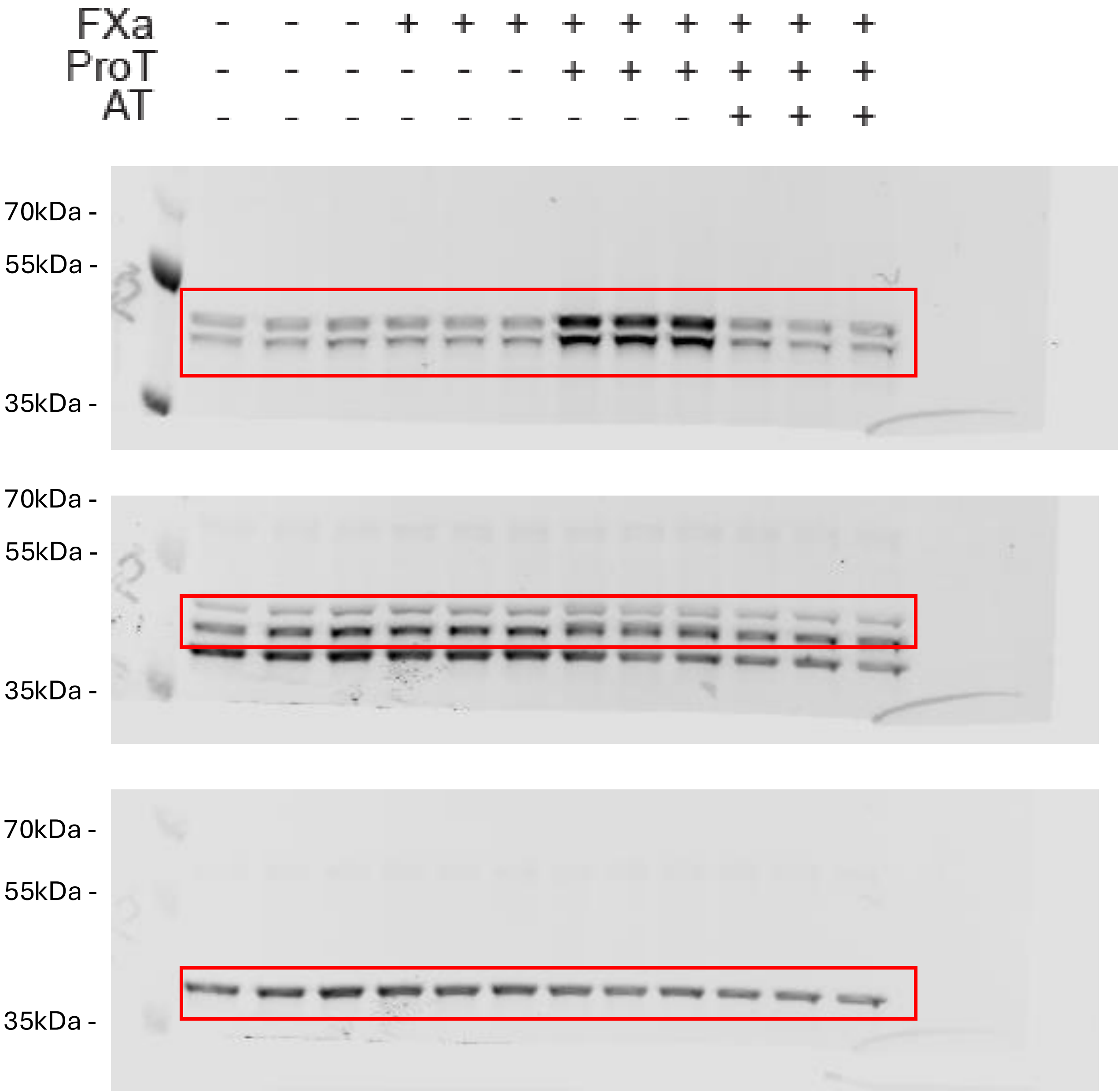

α-phospho-Erk1/2 (P-Erk, thr202/tyr204; Cell Signaling, 9101)

α-Erk1/2 (T-Erk, Cell Signaling, 9102)

α-GAPDH (Cell Signaling, 2118)

Crop

Full unedited blot for HS3ST1 $^{-/-}$ in **Figure 10D**

WT cells

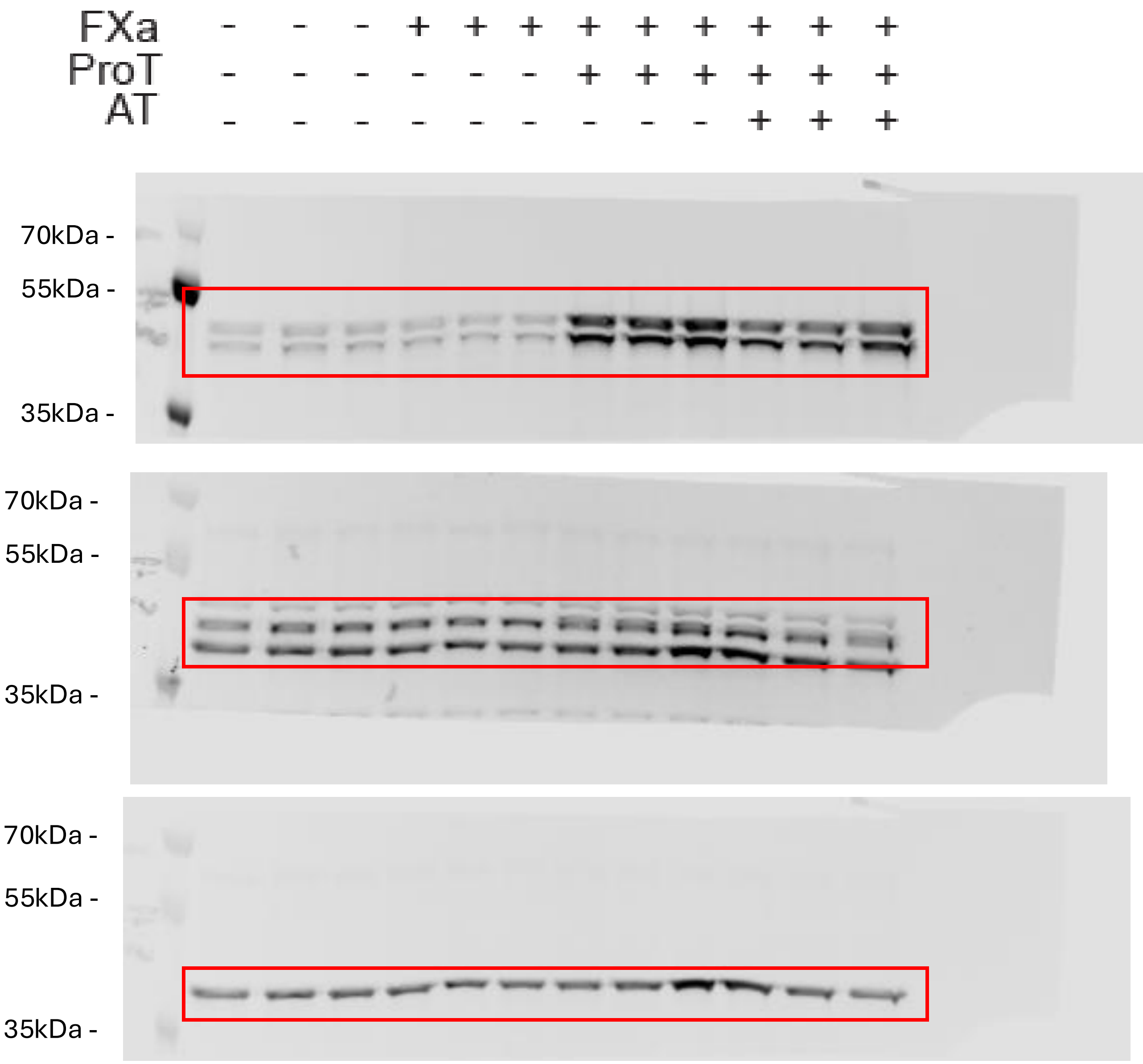

α-phospho-Erk1/2 (P-Erk, thr202/tyr204; Cell Signaling, 9101)

α-Erk1/2 (T-Erk, Cell Signaling, 9102)

α-GAPDH (Cell Signaling, 2118)

Crop

Full unedited blot for KRAS in Supplemental **Figure 5C**

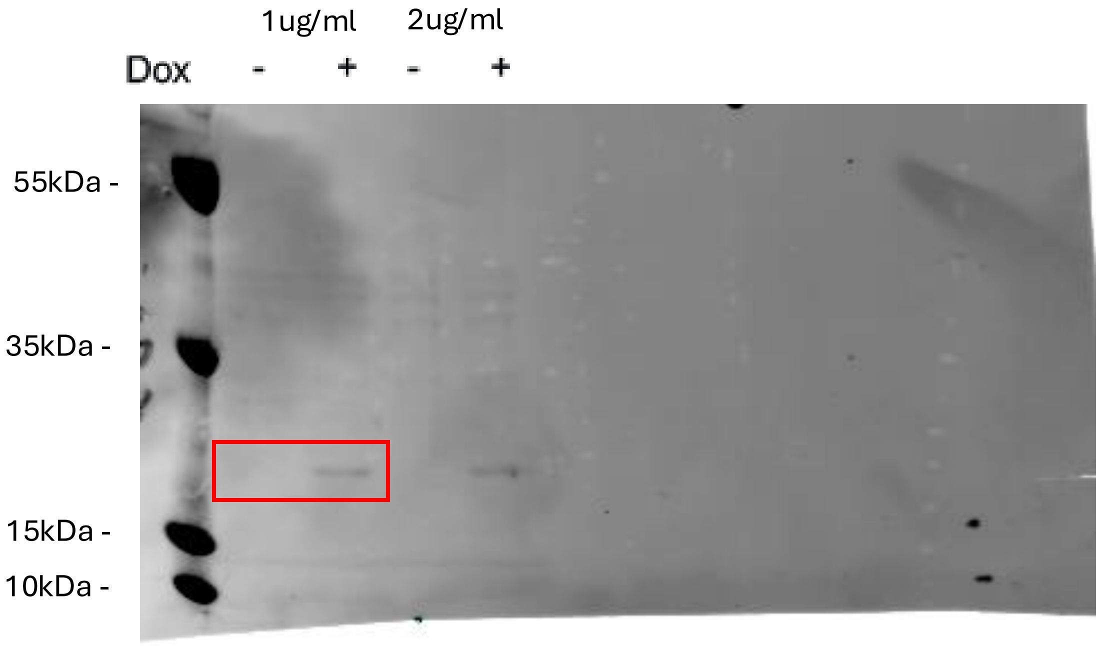

α- KRasG12D (Cell Signaling, 14429)

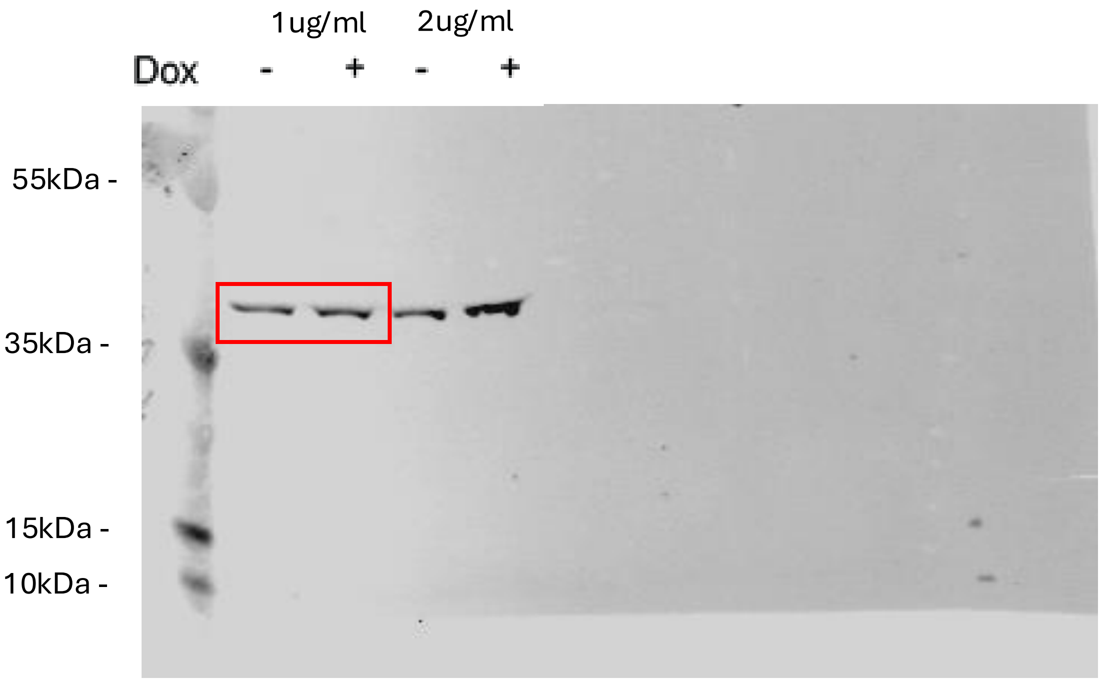

α-GAPDH (Cell Signaling, 2118)

Crop
